# Transcriptional landscape of mitochondrial electron transport chain inhibition in renal cells

**DOI:** 10.1007/s10565-023-09816-7

**Published:** 2023-06-23

**Authors:** Giada Carta, Wanda van der Stel, Emma W. J. Scuric, Liliana Capinha, Johannes Delp, Susanne Hougaard Bennekou, Anna Forsby, Paul Walker, Marcel Leist, Bob van de Water, Paul Jennings

**Affiliations:** 1 Division of Molecular and Computational Toxicology, Vrije University Amsterdam, Amsterdam, the Netherlands; 2 Division of Drug Discovery and Safety, Leiden Academic Centre of Drug Research, Leiden University, Leiden, the Netherlands; 3 In Vitro Toxicology and Biomedicine, Department inaugurated by the Doerenkamp‑Zbinden Foundation, University of Konstanz, Konstanz, Germany; 4 Section for Advice, Danish Patient Safety Authority, Randers, Denmark; 5 Department of Biochemistry and Biophysics, Stockholm University, Stockholm, Sweden; 6 Cyprotex Discovery Ltd., Alderley Park, Macclesfield, Cheshire UK

**Keywords:** Mitochondria, In vitro, Renal, Stress pathway, Transcriptomic

## Abstract

Analysis of the transcriptomic alterations upon chemical challenge, provides in depth mechanistic information on the compound’s toxic mode of action, by revealing specific pathway activation and other transcriptional modulations. Mapping changes in cellular behaviour to chemical insult, facilitates the characterisation of chemical hazard. In this study, we assessed the transcriptional landscape of mitochondrial impairment through the inhibition of the electron transport chain (ETC) in a human renal proximal tubular cell line (RPTEC/TERT1). We identified the unfolded protein response pathway (UPR), particularly the PERK/ATF4 branch as a common cellular response across ETC I, II and III inhibitions. This finding and the specific genes elaborated may aid the identification of mitochondrial liabilities of chemicals in both legacy data and prospective transcriptomic studies.

**Graphical abstract:**

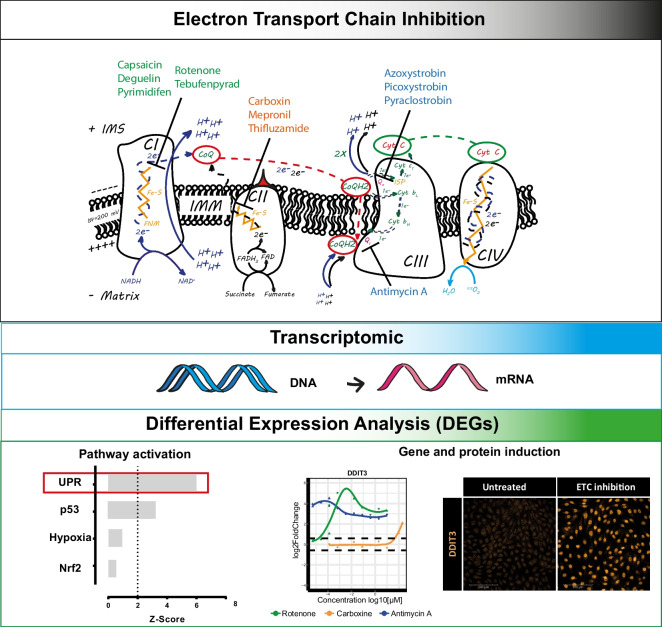

**Supplementary Information:**

The online version contains supplementary material available at 10.1007/s10565-023-09816-7.

## Introduction

The increasing demand of new chemical substances in the fields of agriculture, cosmetics, food and pharmaceutical industries of the last century, has stimulated extra efforts to introduce improved technologies to evaluate potential hazards to humans and the environment. Although, still extensively used, traditional animal-based assays may in certain cases be less relevant to the human situation (Capinha et al. 2021). The principle of the three Rs, proposed by Russel and Burch more than 60 years ago and widely accepted in the scientific community (Russell and Burch 1959), have set the basis for the development of new approach methodologies (NAMs) for next generation risk assessment of chemicals and for regulatory purposes. NAMs have been defined in the EPA’s Toxic Substances Control Act program as “a broadly descriptive reference to any technology, methodology, approach, or combination thereof that can be used to provide information on chemical hazard and risk assessment that avoids the use of intact animals” (US EPA 2019), the most representative being: in silico tools such as quantitative structure–activity relationship QSAR and read-across to estimate effect and doses, in vitro assays to determine mechanisms of action and point of departure of toxicity (PoD), in vitro toxicokinetic for the in vitro-in vivo extrapolation of data (IVIVE) and computer models for the integration of in silico, in vitro and existing toxicology data.

Part of the in vitro sphere of NAMs are the “omics” technologies, in which context, transcriptional evaluation of chemical induced toxicity can provide in depth mechanistic information (Wilmes et al. 2013), including mode of action, to help identify and/or quantify adverse outcome pathways (van der Stel et al. 2021), specific pathway activation/modulation in response to cellular stress and pathway of toxicity (Jennings et al. 2012). Moreover, with the advent of cost-efficient methodologies for transcriptional assessment, such as TempO-Seq assay, high throughput transcriptomic as tool for risk assessment of chemicals became feasible (Limonciel et al. 2018).

In the last decade, evidence has accumulated for a repositioning of chemical induced mitochondrial stress for the development of several toxicologically related adverse outcomes in human populations (Dykens and Will 2008; Nadanaciva and Will 2009; Vuda and Kamath 2016). Beyond classical mitochondrial-specific assays which include fluorescent dyes and oxygen consumption rates, identification of transcriptional changes associated with mitochondrial impairment, could provide hallmarks for the detection of mitochondrial liabilities of drugs.

In the current study, we investigated the transcriptional landscape of mitochondrial toxicity upon cellular exposure to inhibitors of ETC using RPTEC/TERT1 as *in vitro* model. The human RPTEC/TERT1 cell, is a non-cancerous telomerase immortalised cell, originating from a human renal proximal tubule cell (Wieser et al. 2008). These cells become fully contact inhibited, where upon glycolysis is downregulated and oxidative phosphorylation is utilised to fuel cellular processes including water and solute transport from the apical side to the basolateral side of the cells (Aschauer et al. 2013; Wilmes et al. 2015). In addition RPTEC/TERT1 cells when differentiated exhibit an extremely stable phenotype and transcriptomic profile (Aschauer et al. 2013) and exhibit high rates of basal and maximal respiration.

We investigated the effects of acute exposure (24 h) to a range of concentrations of 13 direct inhibitors of complex I (CI-NADH:ubiquinone oxidoreductase), complex II (CII-succinate dehydrogenase) or complex III (CIII-cytochrome *bc*_*1*_ complex) of the ETC on contact inhibited RPTEC/TERT1. Oxygen consumption rates were measured and transcriptomic profiles (TempO-Seq with 2907 transcripts) were generated and analysed.

## Material and methods

### Chemicals

All test compounds were purchased from Merck at one site (JRC, Ispra, Italy) and distributed to the testing laboratories. The catalogue no’s are Capsaicin (M2028), Deguelin (D0817), Fenpyroximate (31684), Pyrimidifen (35999), Rotenone (R8875), Tebufenpyrad (46438), Carboxin (45371), Mepronil (33361), Thifluzamide (49792), Antimycin A (A8674), Azoxystrobin (3167), Cyazofamid (33874), Picoxystrobin (33568), Pyraclostrobin (33696). Additional compounds included: CDDO-me (Selleckchem, S8078), tunicamycin (Tocris Bioscience, 3516) and sodium (meta) arsenite (Sigma-Aldrich, S7400). Test compounds stock solutions between 10 and 100 mM were made in dimethyl sulfoxide (DMSO) and stored at – 20 °C or – 80 °C until use. Treatment solutions were prepared freshly from DMSO stocks for each experiment and the final concentration of DMSO in the systems was 0.1% (v/v).

### Cell culture

The human renal proximal tubule derived cell line RPTEC/TERT1, is a non-cancerous cell line which was immortalised by introduction of the catalytic unit of human telomerase (hTERT) (Wieser et al. 2008). Cells were obtained under licence from Evercyte GmBH, Vienna Austria. RPTEC/TERT1 grow in a monolayer and after reaching confluency become contact inhibited, enter cell cycle arrest and differentiate into a transporting epithelium (Aschauer et al. 2013). RPTEC/TERT1 at passage number between 72 and 95 were routinely cultured in 10 cm dishes (Sarstedt, 83.3902) at 37 ˚C in a 5% CO_2_ humidified atmosphere. Cells were fed every second to third day with medium containing 1:1 mixture of Dulbecco’s modified Eagle’s medium (DMEM, no glucose, Invitrogen, 11,966) and Ham’s F-12 nutrient mix (Invitrogen, 21765), with a final concentration of glucose 5 mM and sodium pyruvate 1 mM, supplemented with 2 mM glutamax (ThermoFisher, 350500038), 5 µg/L insulin, 5 µg/L transferrin and 5 ng/L sodium selenite (Sigma-Aldrich, I1884), 100 U/mL penicillin and 100 µg/mL streptomycin (Merck, P4333), 10 ng/mL epithelial growth factor (Merck, E9644), 36 ng/mL hydrocortisone (Merck, E9644) and 0.5% foetal bovine serum (Gibco, 10,720–106). For experiments cells were plated in required format plate, allowed to become contact inhibited and fed 24 h prior to treatment exposure.

### Oxygen consumption rates with Seahorse XFe96 Bioanalyzer

RPTEC/TERT1 cells were seeded onto Seahorse XF96 V3 PS Cell Culture Microplates (Agilent, 101085–004) at the density of 25,000 cells/well and allowed to differentiate for a minimum of 2 weeks. After differentiation cells were treated for 24 h with a wide range of concentration of CI, CII and CIII inhibitors previous to the mitostress assay performed as previously described in van der Stel et al. (van der Stel et al. 2020). Upon assay completion, measured basal and maximal oxygen consumption rates (OCR) responses were extrapolated by subtracting the average of the positive control response (treatment with a mixture of antimycin A and Rotenone to shut down the ETC), to exclude the non-mitochondrial respiration and setting as upper asymptote (100%) the average of all negative controls’ basal response (0.1% DMSO vehicle control). Data was further expressed as percentage of at least two non-effective concentrations (if applicable) to overcome random variations not linked to the biological effect and decrease the uncertainty around the benchmark concentration (BMC) calculations (e.g. inhibitory concentration of 10%, IC10) (Krebs et al. 2018).

### Immunofluorescence

RPTEC/TERT1 cells were seeded and differentiated into CellCarrier ultra-black 96-well plates (PerkinElmer, 6055302) and treated for 24 h with one representative concentration (see Table 2) of each of the CI, CII, and CIII inhibitors of the electron transport chain. After 24 h cells were fixed with 4% PFA for 20 min, permeabilised with 0.1% Triton X-100 for 10 min and blocked with 5% BSA for 1 h. Samples were incubated for 2 h at RT with rabbit α-*DDIT3* primary antibody (MyBiosource, MBS9405289, 1:500), followed by incubation for 1 h with α-rabbit Alexa Fluor™ 546 secondary antibody (ThermoFisher, A10040, 1:1000) and Hoechst 33342 (ThermoFisher, H3570, 1:10.000) for 20 min. Samples were imaged using the Operetta CLS high content imager (PerkinElmer) with × 40 water objective in confocal mode. Images were gathered with Harmony software 4.8.

DDIT3 protein quantification analysis was performed with Harmony software 4.8 using the following analysis pipeline: (1) nuclei were counted based on the Hoechst 33342 channel. (2) The mean of intensity signal of the Alexa Fluor™ 546 (DDIT3) inside each nucleus was calculated. (3) An intensity threshold for positivity was set based on accumulation of DDIT3 in 0.1%DMSO control samples nuclei. (4) For each treatment condition the number of positive nuclei above the set threshold was calculated. (5) Results were defined as percentage of positive nuclei over the total number of nuclei in each sample.

### Western blotting

RPTEC/TERT1 cells were seeded and differentiated into 6-well plates (Greiner, CELLSTAR®, CLS3516) and treated for 24 h with one representative concentration (see Table 2) of each of the CI, CII, and CIII inhibitors of the electron transport chain. After 24 h, cells were washed in ice cold PBS and scraped in 150 µL RIPA buffer (Sigma, 0278) containing 1% protease inhibitor cocktail (Sigma, P8340). Protein fractions were centrifuged at 10.000 × *g*, pellets discarded, and lysates stored at – 20 °C prior to use. Protein concentration was determined with the Pierce^tm^ BCA Protein assay kit (Thermo scientific, 23227). Western blots were performed using self-made 12% SDS-PAGE gels and 21.4 µg of protein containing 50 mM DTT were loaded in each well. Running buffer consisted in 190 mM glycine, 25 mM Tris-base and 0.1% SDS, electrophoresis was run at 100 V for 10–15 min and then at 200 V for 45–60 min. Proteins were transferred onto a methanol activated PVDF membranes (GE Healthcare Life Sciences, USA) using a transfer buffer containing 192 mM glycine and 25 mM Tris-base and 20% methanol for 1 h at 600 mA. Membranes were washed in PBS-T, blocked for 1 h in PBST with 5% skimmed milk and incubated either for 1 h at RT or overnight at 4 °C first with the primary antibodies mouse α-actin (Santa Cruz, sc-47778, 1:1000) and mouse α-*HMOX1* (Abcam, ab13248, 1:1000). After three washes in PBS-T, membranes were incubated with α-mouse-HRP secondary antibody (Biorad, 1706516, 1:10.000) for 1 h at RT. After three washes in PBS-T, the chemiluminescent substrate ECL (Pierce, 32106) was added to the membranes for 5 min before imaging with Sapphire Biomolecular Imager (Azure Biosystems, USA). Actin and *HMOX1* detection were carried out at different times on the same membrane due to antibody specie incompatibility.

### TempO-Seq experiments

RPTEC/TERT1 cells were seeded and differentiated onto 96-well cell culture microplate (Greiner, CELLSTAR®, 655180) and treated for 24 h with a wide range of concentrations of CI, CII and CIII inhibitors of the electron transport chain. After 24 h, treatments were replaced with 80 µL of lysis buffer (Byospider, 200001) and froze at – 80 °C until shipping. Transcription of a customed panel of 3256 human genes (Table S1), representing the overall cellular activity and toxic responses, was measured using the targeted sequencing approach provided by BioClavis, TempO-Seq (Limonciel et al. 2018). The gene list includes the “S1500 +  + ” gene list established by the EUToxRisk consortium (https://www.eu-toxrisk.eu), originally set in the U.S. Tox21 Federal collaboration (Mav et al. 2018), plus a set of additional probes relevant in toxic treatments relative to the list of tissues used in the consortium. Two experiments were conducted including treatments with different concentrations of the same compounds (Table S1), for the two experiments, two different probe panels were employed, referred to in this manuscript as panel v2.0 and panel v2.2 for the first and second experiment respectively. The two panels show some differences in the probes used for targeting the same genes (a detailed comparison is provided in Table S2).

Upon TempO-Seq assay completion, generated FASTQ files are aligned by the manufacturer and relative output files were delivered as row count tables indicating the number of read counts of each probe in each sample for the two datasets.

### Single dataset data analysis pipeline

Analysis was conducted in RStudio version 1.2.5033, as quality control was excluded from the analysis samples with library size below 200.000. Filtered raw counts tables, were further processed using the DESeq2 v1.28.1 package (Love et al. 2014).

The two datasets were analysed separately running the DESeq2 independently for each treatment and its relative control, using the design formula below:

$$\begin{array}{c}\mathrm{DESeqDataSetFromMatrix}(\mathrm{countData }=\mathrm{ count table},\\ \mathrm{ colData }=\mathrm{ meta table},\\ \mathrm{ design }= \sim \mathrm{ treatment}\end{array}$$

Differential expression analysis of genes, for each condition, was achieved by pairwise comparison between treatment condition and relative vehicle control group (0.1% DMSO), each of which included three biological replicates.

The DESeq2 method takes a table of raw counts as input and perform an internal normalisation calculating the geometric mean for each gene across all samples to be used to correct the raw read counts for the size of the library and generate a normalised read counts table. Moreover, it uses shrinkage estimators for dispersion and fold change calculations (Anders and Huber 2010) before testing for differential gene expression. A description of parameters generated with DESeq2 analysis for each treatment is summarised in Table 1.

**Table 1:** DESeq2 analysis outcome parameters per gene for each treatment condition (Love et al. 2014)

Parameter	Description
Ctr. read counts	Normalised read counts of 0.1% DMSO control samples
Treat. read counts	Normalised read counts of treatment samples
baseMean	Average of normalised counts taken over all samples of compared groups
log2FoldChange	log2 fold change of treatment group over control group
lfcSE	Standard error of the log2Fold change
stat	Wald statistic
pvalue	Wald test *p* value
padj	Benjamini–Hochberg adjusted *p* value

After normalisation with DESeq2, data was filtered for baseMean > 10, to diminish low read count related noise and a significance cut-off was applied of padj < 0.05 and abs log2 fold change > 0.585.

### Class-specific data analysis pipeline

For the class comparison analysis, a new DESeq2 analysis was run for each of the three classes of inhibitors (CI, CII and CIII). Class-specific analysis was obtained by using as countData input file the overall class-specific response consisting in the row read counts of one concentration per compound and relative controls. Representative concentrations per compound correspond to the highest response in terms of significantly differentially expressed genes (DEGs) obtained in the compound-specific analysis (Fig. 2, Table 2) and are represented in one of the two experiments depending on the compound. Due to the discrepancy between the two gene panels used in different experiments, different probes between the two panels were excluded for this analysis, unless giving similar response in the two datasets (Figure S1). The new obtained list includes 2907 genes (3184 probes) and will be referred to as *class-specific gene list* (Table S3).

**Table 2:** Toxic profile of reference concentrations of ETC inhibitors including relative in vivo LD50s and IVIVE of equivalent administered doses in humans

Complex inhibited	Compound name	Acute toxicity category	Reference concentration (µM)	LD50 mg/kg (acute oral/rats)	EAD50 mg/kg/dose (human)
CI	Capsaicin	II	1.00E + 02	148.10	3.12E + 01
CI	Deguelin	NA	8.00E − 02	NA	3.14E − 02
CI	Fenpyroximate	II	1.60E − 02	245.00	5.99E − 03
CI	Pyrimidifen	II	1.60E − 02	115.00	5.84E − 03
CI	Rotenone	I	6.40E − 04	70.75	2.38E − 04
CI	Tebufenpyrad	II	1.00E + 01	202.00	2.98E + 00
CII	Carboxine	III	2.00E + 02	2588.00	8.31E + 01
CII	Mepronil	IV	5.00E + 02	> 10,000	1.30E + 02
CII	Thifluzamide	IV	2.70E + 02	> 6500	1.28E + 02
CIII	Antimycin A	II	2.56E − 05	323.50	NA
CIII	Azoxystrobin	IV	1.00E + 01	> 5000	5.54E + 00
CIII	Picoxystrobin	IV	2.00E + 00	> 5000	8.31E − 01
CIII	Pyraclostrobin	IV	4.00E − 01	> 5000	1.49E − 01

Data represent 24 h exposure of a single dose of the drug

As the class-specific countData file, includes row counts generated in the two different experiments, the new DESeq2 analysis was run for each of the three classes using the adjusted design formula below, where the exp_*ID*_ refers to the corresponding experiment:

$$\begin{array}{c}\mathrm{DESeqDataSetFromMatrix}(\mathrm{countData }=\mathrm{ class specific count table},\\ \mathrm{colData }=\mathrm{ class specific meta table},\\ \mathrm{design }= \sim {\mathrm{exp}}_{\mathrm{ID}}+\mathrm{ class}\end{array}$$

The designed formula, followed by testing of fold changes due to class, will account for changes in counts due to batch effect, and give marginal effect of the class across all levels (experiments).

After normalisation with DESeq2, data was filtered for baseMean > 10, to diminish low read count related noise and a significance cut-off was applied of padj < 0.05 and abs log2 fold change > 0.585.

### Pathway analysis

To find out which pathways are enriched with regulated genes, an over representation analysis (ORA) of the datasets was performed using a double approach with the two bioinformatic platforms PathVisio (Department of Bioinformatics at Maastricht University, NL), and Ingenuity Pathway Analysis-IPA (QIAGEN Inc., https://www.qiagenbioinformatics.com/products/ingenuitypathway-analysis).

### Pathway analysis with PathVisio

Pathways statistical analysis and pathway data visualisation diagrams were generated using the software PathVisio 3.0.0 + (Kutmon et al. 2015). Activated pathway identification in PathVisio relies on genes ORA. The software blasts the imported expression data against a collection of pathways collectively called the WikiPathways database (*.gpml files) (wikipathways.org) (Slenter et al. 2018), bridging the expression data to the genes in the pathway with the specie-specific identifier mapping database Hs_Derby_Ensembl_91.bridge. The PathVisio statistic module, computes two statistical analysis: the *Z*-score and the permuted *p* value.

i) The *Z*-score indicates the ratio of genes meeting given statistical criteria with the total genes present in each pathway of a given database. It is used to rank the over-representation analysis and is calculated by a standard statistical test under the hypergeometric distribution with the following equation: $Z-\mathrm{score}=\frac{(\mathbf{r}-\mathbf{n}\frac{{\varvec{R}}}{{\varvec{N}}})}{\sqrt{\mathbf{n}\left(\frac{{\varvec{R}}}{{\varvec{N}}}\right)\left(1-\frac{{\varvec{R}}}{{\varvec{N}}}\right)\left(1-\frac{{\varvec{n}}-1}{{\varvec{N}}-1}\right)}}$, where *N* is the total number of measured genes in the experiment, *R* is the number of genes meeting the selection criteria, n is the total number of genes in a specific pathway and r is the number of genes meeting the selection criteria in that specific pathway. A *Z*-score > 2 indicates that significantly more genes than expected are changed in that pathway, a *Z*-score = 0 indicates that the distribution of changed genes in the pathway is the same as in the complete dataset and a *Z*-score <  − 2 indicates that less genes than expected meet the selection criteria meaning that the pathway in question is very stable (Kutmon et al. 2015).
ii) The permuted *p* value is used as statistical value to reject the null hypothesis.

For pathway analysis in this study, cut-offs of significance of padj < 0.05, abs log2 Fold Change > 0.585 were applied to expression data prior to ORA, and an in-house built database of pathways was used. The used pathway database consists of 109 pathways, 94 of which were included from the WikiPathways database (representing the overall cellular physiological activity) and 15 were developed in-house (representing mitochondrial activity and stress response pathways). The detailed list of pathways and relative node list are listed in Table S4. Further applied cut-off of significance included permuted *p* value < 0.05 and abs *z*-score > 2.

### Core analysis with IPA

A gene/protein expression core analysis was performed in the IPA software. Two main statistical analysis are computed in IPA: the overlap *p* value and an activation *Z*-score. The two measures are calculated independently from each other and provide insights into different aspects of the analysis.

i) The p-value of overlap is used to reject the null hypothesis, for which the overlap of genes in a given dataset with the ones in a particular process (pathway, function, disease etc.) is due to chance alone, it is calculated using the right-tailed Fisher’s exact test (*p* value) and can be implemented with the Benjamini-Hochberg (*q* value) correction for false positives. It is generally considered a non-random association an overlap with *p* value < 0.05.
ii) The activation *z*-score is used predict the activation or inhibition state of the overlap between the dataset and what would have been expected according to the Ingenuity Knowledge Data (collection of data derived from different sources such as scientific literature, public databases and experimental data) using the following formula:

$z-\mathrm{score }(r)=\frac{\sum_{v\in \tilde{O} }{w}_{R }\left(r,v\right){s}_{R }\left(r,v\right){s}_{D}\left(v\right)}{{\left(\sum_{v\in \tilde{O} }{\left[{w}_{R }\left(r,v\right)\right]}^{2}\right)}^{1/2}}$, for details refer to (Krämer et al. 2014).

A *z*-score ≥ 2 indicates a prediction of activation and a *z*-score ≤ − 2 indicates a prediction of inhibition.

### Pathway analysis with IPA

The statistical parameters of the core analysis are computed for Pathway analysis in IPA by associating the molecules of the given input dataset with the ones present in each of the pathways included in the IPA pathways list, originated from the Ingenuity Knowledge Base. For specific compounds pathway analysis, a cut-off of *p* value < 0.05 and abs i-score > 2 was applied, and only pathways induced in more than 4 compounds were taken in consideration. For class-specific pathway analysis, a cut-off of *p* value < 0.05 and abs *z*-score > 2 was applied, and only pathways including a minimum of 20% of the total genes changed were taken in account.

### Upstream regulator analysis with IPA

The upstream regulators analysis performed in IPA, is based on expected effects between transcriptional regulators and their target genes according to the Ingenuity Knowledge Data for the identification of key small molecules triggering the cascades of events leading to the observed effect.

The overlap *p* value calculates, using a Fisher’s exact test, whether the overlap between genes in the test dataset and genes regulated from a given transcription factor is statistically significant, the activation *z*-score is used to find probable regulating molecules and to deduce the activation state of a putative transcription factor based on what would have been expected according to the Ingenuity Knowledge Database.

For a prediction to be made a certain number of downstream genes changed in the test dataset must overlap the transcription factor downstream gene list. For example, if the activation of transcription factor A is expected to induce the upregulation of a set of downstream genes in the dataset, it is observed an upregulation of those genes, transcription factor A is predicted to be activated. Whereas if in the dataset it is observed a downregulation of the same genes, transcription factor A is predicted to be inhibited. The same is true for expected downregulation of downstream genes. Cuts-off of significance for upstream regulator analysis include abs. activation *z*-score > 2 and BH corrected p-value of overlap.

### Dose response analysis

To estimate the concentration at which test compounds exposure starts inducing a change in gene expression (PoD) a dose–response modelling was performed using the software BMDExpress 2 (Yang et al. 2007; Phillips et al. 2019). Two different analyses were performed for the two different experiments.

Benchmark Dose (BMD) analysis consisted in two steps:

1. Prefiltering of data to eliminate noise by selecting only probes with a statistically significant dose–response. After loading the normalised gene expression response data and application of annotation to probes, expression data was filtered by applying a William’s trend test with a *p* value < 0.05 and abs fold change > 1.5 cut-off.

BMDs retrieval from fitted dose response curves using the Exp5 parametric model.

### IVIVE of reference concentrations of compounds

To ensure the relevance of selected reference concentrations in relation to the in vivo situation, namely, to determine the in vivo toxic effect of tested compounds, we have performed the in vitro-in vivo extrapolation (IVIVE) of used reference concentrations. IVIVE determines the exposure dose that leads to a plasma concentration equivalent to the tested *in vitro* concentrations (Chang et al. 2022). IVIVE was performed with the Integrated Chemical Environment (ICE) (Bell et al. 2017) (publicly accessible at https://ice.ntp.niehs.nih.gov). ICE used in vivo test data, chemical information, in vitro assay data (including Tox21TM/ToxCast™ highthroughput screening data) and in silico model predictions to build a computational workflow for the estimation of the queried in vitro equivalent administered doses (EADs). Along with test reference concentrations, EADs relative to the activity concentrations at cutoff (ACC) of a series of in vitro relevant toxicity endpoints included in the ICE curated high-throughput screening (cHTS) were estimated using the following parameters: (1) default ADME source, which includes experimentally measured values when available and the Open (Quantitative) Structure–activity/property Relationship App (OPERA) prediction when empirical data is lacking. (2) Solve_3comp PK model including liver, gut, artery, vein, lung, and whole-body compartments. This model estimates the *C*_max_ and simulates the oral exposure. (3) Oral exposure route. (4) 24 h exposure interval for 1 dose.

### Class-specific mitotoxic response analysis

To extrapolate a mitochondrial-specific transcriptional response, we have performed a class-specific mitotoxic response analysis by applying the same logic used for the class-specific responses. A new DESeq2 analysis was performed merging representative concentrations of compounds per class. Mitotoxic representative concentrations were determined by a minimum of 40% decrease in OCR in the non-cytotoxic/subtoxic range (max IC25). These included 0.08 µM deguelin, 0.08 µM fenpyroximate, 0.08 µM pyrimydifen, 0.016 µM rotenone for CI and 0.016 µM antimycin A, 2 µM pyraclostrobin for CIII, and excluded capsaicin and tebufenpyrad for CI, all CII inhibitors and azoxystrobin and picoxystrobin for CIII.

### Supplemental material

**Fig. 1:**
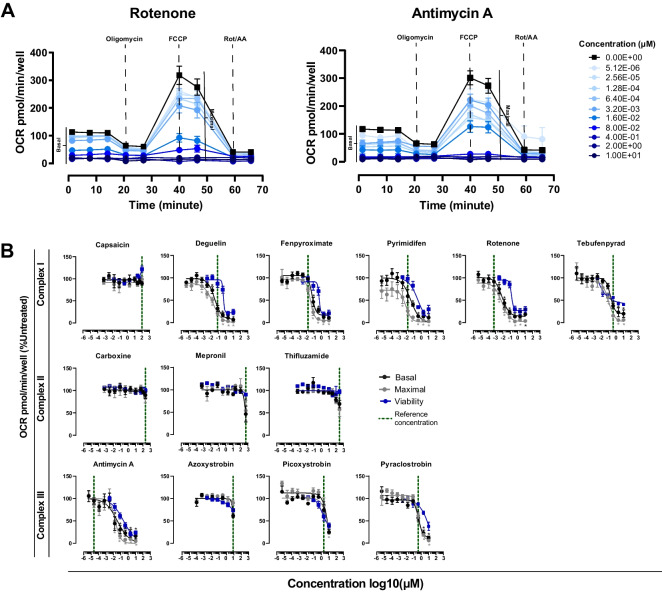
Oxygen consumption rates in treated RPTEC/TERT1. **A **Diagram of the mitostress test using the Seahorse analyzer upon 24 h exposure to a dose range of rotenone and antimycin A as representation of input data for the basal and maximal dose response curves extrapolation. **B** Dose dependent effect on basal (black) and maximal (grey) respiration rates extrapolated from the mitostress assay upon 24 h exposure to a range of concentrations (Table S1) of CI, CII, and CIII inhibitors. Data represent the mean of 3–7 independent experiments ± SEM expressed as percentage of vehicle treated control samples (0.1% DMSO). Statistical significance was computed by one-way ANOVA followed by Dunnett’s multiple comparisons posttest. Asterisks indicate a *p* value < 0.05. Vertical dotted line corresponds to the reference concentrations of compounds inducing the highest number of DEGs and used in the transcriptomic analysis. Blue slopes indicate the dose dependent effect of compounds on viability (van der Stel et. al. 2021)

In the supplemental material are provided: (1) a table of compounds and relative concentrations used in the two TempO-Seq experiments (Table S1). (2) A table with the list of genes and probes included in panel v 2.0 and panel 2.2 and relative gene information (Table S2). (3) A table with the list of genes included in the class-specific analysis (Table S3) and criteria of selection for different probes targeting the same gene in the two panels (Figure S1). (4) A table indicating the pathways included in the PathVisio pathway analysis database, including the gene lists of the in house drawn pathways (Table S4). (5) A table containing raw read counts of experiments 1 and 2 with relative annotation files (Table S5). (6) A table containing the significant class-specific induced gene lists indicated in Fig. 3B: the intersect gene list, the list of genes changed by two of the three classes, and the lists of CI, CII and CIII unique induction. All lists include information about FoldChange, baseMean and *p*-adj for the three classes CI, CII and CIII (Table S6). (7) Viability and OCR responses to a range of concentrations of the UPR activator tunicamycin (Figure S2A). (8) PatVisio pathway analysis of the intersect gene list (318 genes commonly changed by CI, CII and CIII inhibition) is provided in Figure S2B. (9) The heatmap of the class-specific p53 induction, comparing the log2 fold change over control of genes part of the p53 pathway induced by class I, II and III (Figure S2C). The analysis results of the class-specific mitotoxic response are provided in Figure S3, including a reduced version of Fig. 1 depicting selected compound and concentrations (A), volcano plots of CI and CIII Mitotoxic response (B) and ORA analysis performed with PathVisio (C). (10) Heatmaps of shared genes between the two bioinformatic platforms (IPA, PathVisio) for the cholesterol biosynthesis and eIF2 signalling-Ribosomal proteins pathways (Figure S4A, C). (11) Graphical representation in PathVisio of the UPR pathway induced by CII and CIII inhibition is provided in Figure S5. (12) Representative images of DDIT3 immunofluorescence in RPTEC/TERT1 upon treatment with inhibitors not represented in Fig. 8B are shown in Figure S6.

## Results

### Effect of ETC’s complexes inhibition on mitochondrial respiration

To determine the level of direct mitochondrial perturbation, basal and maximal OCR were measured upon 24 h exposure with a wide range of concentrations of CI, CII and CIII inhibitors of the ETC in RPTEC/TERT1 cells. All compounds, except for CI inhibitor capsaicin and CII inhibitor carboxine decreased basal and maximal OCR in a concentration dependent manner (Fig. 1). CI inhibitors deguelin, fenpyroximate, pyrimidifen showed an impaired respiration (basal, maximal or both) before the onset of cytotoxicity, whereas for CIII inhibitors antimycin A and pyraclostrobin exhibit 60% and 40% decrease in basal and maximal OCR respectively at concentrations that decrease viability of about 10%. An impairment of mitochondrial respiration at non-cytotoxic concentrations suggests a direct effect of test chemicals on mitochondrial function possibly leading to cellular death. CII inhibitors mepronil and thifluzamide impair OCR only at highest tested concentrations and in the non-cytotoxic range, however CII tested concentrations were several orders of magnitude higher than the other inhibitors suggesting that the reduced OCR can be due to an off-target effect (Fig. 1). This is in line with experiments conducted in permeabilised cells and available in a previous publication from this group (van der Stel et al. 2020), where observed inhibitory concentrations of CII inhibitors were 5 µM, 50 µM and 15.8 µM for carboxine, mepronil and thifluzamide respectively, highlighting the physiological relevance of CII inhibition, negligible in the presence of functional CI. The remaining ETC inhibitors, tebufenpyrad, azoxystrobin and picoxystrobin showed a decrease in OCR starting at cytotoxic concentrations, indicating a reduction in OCR probably due to a reduced number of live cells. However, CI and CIII observed inhibitory concentrations reflect those characterised in the complex-specific respirometry (permeabilised cells), corroborating the specificity of the effects of tested compounds on mitochondrial respiration and possibly explaining the non-effect of capsaicin which start its inhibitory activity at a higher concentration (158 µM) than the maximal tested in this study (100 µM). Collectively, the data display the mitochondrial toxic profile of tested compounds.

### Differentially expressed genes vs mitochondrial perturbation and cytotoxicity

**Fig. 2:**
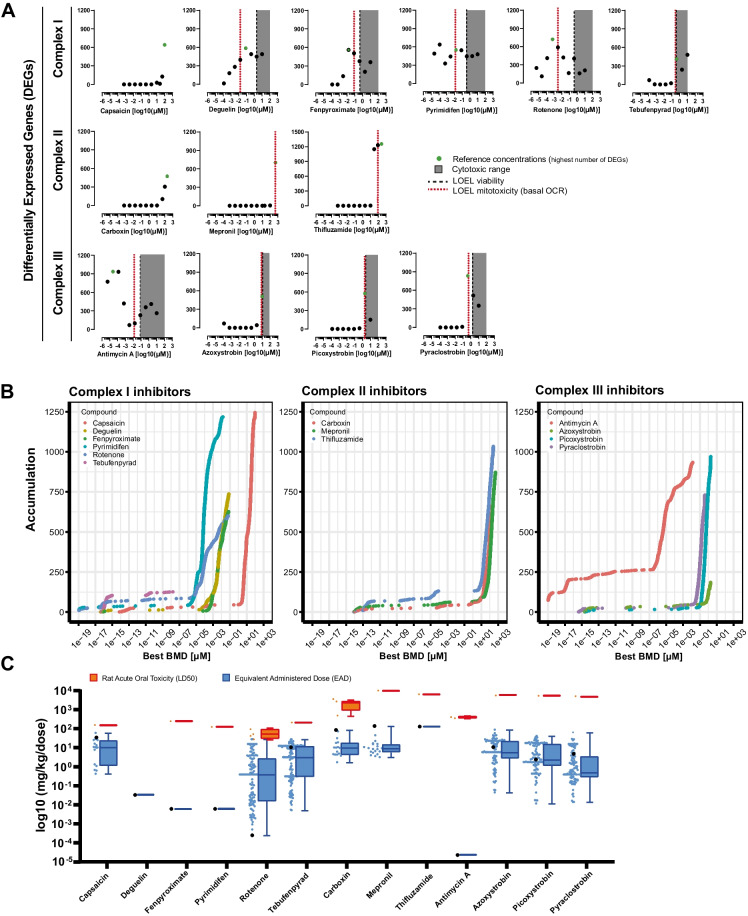
Differentially expressed genes. **A** Number of significantly differentially expressed genes per treatment after 24 h exposure of RPTEC/TERT1 cells with a range of concentrations of CI, CII, and CIII inhibitors of the ETC. Criteria of significance: padj < 0.05, abs log2 fold change > 0.585 and base mean > 10. In grey the cytotoxic range, starting at the black dotted line, defined by the LOEL of viability response. Red dotted lines indicate the point of departure of mitochondrial toxicity, defined by LOEL in basal OCR at the same experimental conditions. **B** Accumulation plots of Best BMDs computed in BMDExpress2 induced by treatment of RPTEC/TERT1 cells with a range of concentrations of CI, CII and CIII inhibitors of the ETC 24 h. **C** IVIVE of equivalent administrated doses corresponding to reference concentrations of test compounds performed with the ICE web resource. EADs or reference compounds (black dots) are compared to the in vivo LD50s (orange dots) and the EADs at ACC of several in vitro toxicological end points part of the ICE database (blue dots)

To picture the general effect of treatments on genes transcription in RPTEC/TERT1 cells and correlate the changes in gene expression to cellular and mitochondrial toxicity, we calculated the total number of significantly differentially expressed genes (DEGs) upon 24 h exposure of a range of concentrations (Table 2) of CI, CII and CIII inhibitors. All compounds exhibit changes in DEGs before the onset of cytotoxicity, suggesting a specific transcriptional response to the insult. However, whereas treatment with most CI inhibitors (deguelin, fenpyroximate, pyrimidifen and rotenone) and CIII inhibitor antimycin A induces DEGs in the nanomolar range, treatments with CI inhibitor capsaicin, all CII inhibitors, and the rest of CIII inhibitors showed DEGs induction either at high concentrations or at doses that coincide with the initiation of cellular death indicating a difference in potency between the compounds (Fig. 2A). Transcriptional changes induced at cytotoxic concentrations result less specific when studying the adaptative response to chemical stress, therefore DEGs induction in the cytotoxic range have not been further considered in this study. We further evaluated the link between transcriptional induction and mitochondrial toxicity by comparing DEGs induction with the starting point of mitochondrial insult, represented by the LOEL of basal OCR. Strong compounds inducing DEGs at low doses also decrease mitochondrial respiration at lower concentrations needed to induce toxicity indicating a direct correlation between mitochondrial toxicity and transcriptional response (Fig. 2A, vertical red lines). Such correlation is probably maintained also for all the other inhibitors inducing mitochondrial impairment but with a lower degree of potency. It must be noticed that induction of DEGs and mitochondrial insults appear visible upon inhibition of the two CII blockers mepronil and thifluzamide only at high concentrations (hundreds in the µM range) implicating a possible lack of specificity.

Perturbations of biological processes induced by chemical application, are intrinsically dose dependent. To establish a correspondence between doses of tested chemicals and changes in gene expression levels, a BMD modelling was performed in DMBExpress2. BMD methods allow for estimation of the potency of a chemical in changing biological processes as well as the dose at which such a change can be expected, representing the PoD of gene expression alteration. Predicted BMDs are summarised in accumulation plots, the lower the BMD the higher the potency to induce that specific gene. Compounds driven genes expression accumulation, mirrors the DEGs induction pattern, with antimycin A predicted to accumulate ~ 125 probes at concentration of ~ 1 × 10^−19^. The rapid increase of the accumulation slope, namely the concentration at which most of the changes in gene expression occur, resulted at low concentrations for antimycin A, deguelin, fenpyroximate, pyrimidifen, rotenone A and in high concentration for the other compounds (Fig. 2B).

The relationship between DEGs, OCR and cytotoxicity ranks antimycin A, deguelin, fenpyroximate, pyrimidifen, rotenone as strong compounds exerting their activity before inducing cytotoxicity. Higher deleterious effect of CI and CIII (via Q_i_ site inhibition–antimycin A) inhibition compared to the other inhibitors is corroborated by their gene accumulation at low concentrations. Moreover, in most of the cases changes in transcription initiate at lower concentrations needed to induce changes in other tested endpoints, indicating a higher sensitivity of transcriptomics over the other assays.

To narrow down the number of doses to be further analysed, we defined one reference concentration per compound corresponding to the highest number of induced DEGs outside the cytotoxic range (Table 2). With the awareness that in vitro systems do not recapitulate human physiology and to corroborate the applicability of chosen reference concentrations, we performed and IVIVE using the ICE IVIVE tool. Obtained equivalent doses were compared to EADs at ACC of a series of in vitro toxicity endpoints available in the ICE database as well as to the LD50s in rats orally exposed to test compounds for 24 h (Fig. 2C). All used concentrations resulted lower to those needed to induce mortality in vivo reflecting the relevance of effective doses of test compounds in an in vivo context. Moreover, comparison of reference concentrations with ACCs of other in vitro assays demonstrates the increased sensitivity of transcriptomic in a contest of a specific induction (e.g. induction not related to borderline toxicity) as indicated by rotenone in Fig. 2C.

### Transcriptional evaluation of class-specific ETC inhibition

Exposure to all ETC inhibitors resulted in the induction of DEGs at non-cytotoxic or sub-toxic concentrations and in some cases before mitochondrial insult. To investigate whether inhibition at different levels along the ETC affects the expression of the same genes, we performed a class comparison analysis. Responses of class I (CI), class II (CII) and class III (CIII), representative of the associated respiratory complexes were obtained by combining the responses of all compounds belonging to a class, at reference concentrations, for the generation of class-specific profile (see “Class-specific data analysis pipeline” section for details).

**Fig. 3:**
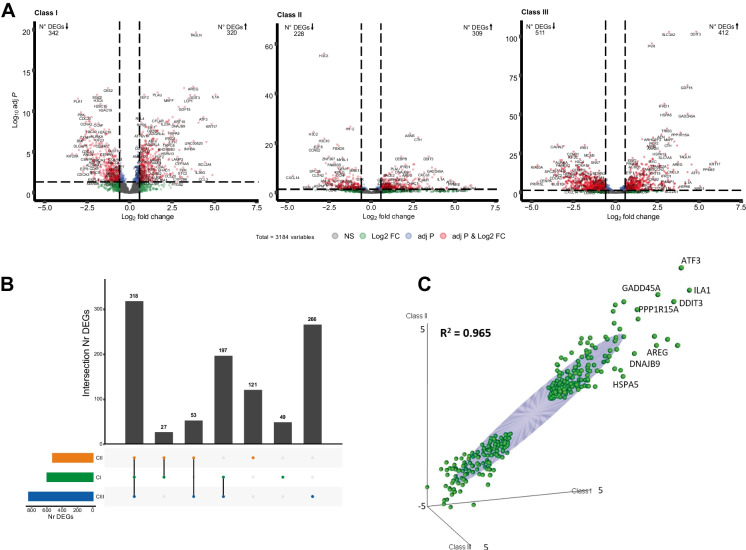
Class-specific induced DEGs. **A** Volcano plots identifying significantly changed genes for the CI, CII, and CIII inhibition. Grey dots: non-significant genes. Green dots: genes with abs fold change over control > 1.5. Blue dots: genes with *p* adjusted value < 0.05. Red dots: genes fulfilling both criteria of significance-abs fold change over control > 1.5 and *p* adjusted value < 0.05. **B** Intersection plot of class-specific significant DEGs. 318 genes are significantly changed by CI, CII, and CIII inhibition. **C** Correlation plot of the fold change of common changed DEGs within the three classes of inhibition (318 genes). Criteria of significance: *p* adj < 0.05, abs fold change > 1.5

Volcano plots indicate a substantial overall activity, in terms of induced or inhibited transcription for the three classes (Fig. 3A). However, comparison in the number of up and down regulated genes among classes, identifies CIII inhibition as having the highest impact with a total of 511 downregulated and 412 upregulated genes. To obtain a general transcriptional signature of ETC inhibition, we wanted to identify genes commonly effected by all ETC inhibitors and genes specifically changed by treatment with one of the classes (Fig. 3B). Three hundred eighteen genes were found to be affected upon exposure to all three classes of inhibitors, we will refer to this list of genes as the intersect gene list. To evaluate whether common induced genes are changed in the same manner, the fold change over control responses of the intersect genes were plotted in a correlation graph (Fig. 3C). The plot underlines a linear correlation, with an *R*^2^ of 0.965, indicating that the three classes change common genes in the same manner. The analysis also identified a list of genes significantly changed by only one class on inhibitors, 49, 121 and 266 for CI, CII and CIII respectively (Fig. 3B, Table S6). However, ORA analysis with PathVisio pathway did not reveal any pathway-specific response. Moreover, considering a baseMean > 50 the strongest response was given by *MMP1* with a log2FoldChange of 1.1 for CI and *SNAI2* with a log2foldchange of 1.8 for CIII, considerably marginal compared to the intersect gene expression. Interestingly, a few unique genes of CII resulted higher in expression (above 2 log2FoldChange), including heat-shock proteins *HSPA1B* (log2FC 3.3) and *HSPA1A* (log2FC 2.35) involved in maintaining proteostasis (Rosenzweig et al. 2019), and *CYP2C19* (log2FC 2.6) involved in xenobiotic metabolism (Sim et al. 2006).

### Pathway enrichment analysis identified a common UPR response for ETC inhibitors

To evaluate specific pathway enrichment amongst altered genes per class and single compound, a statistical ORA analysis was performed using two bioinformatic platforms: PathVisio and IPA.

**Fig. 4:**
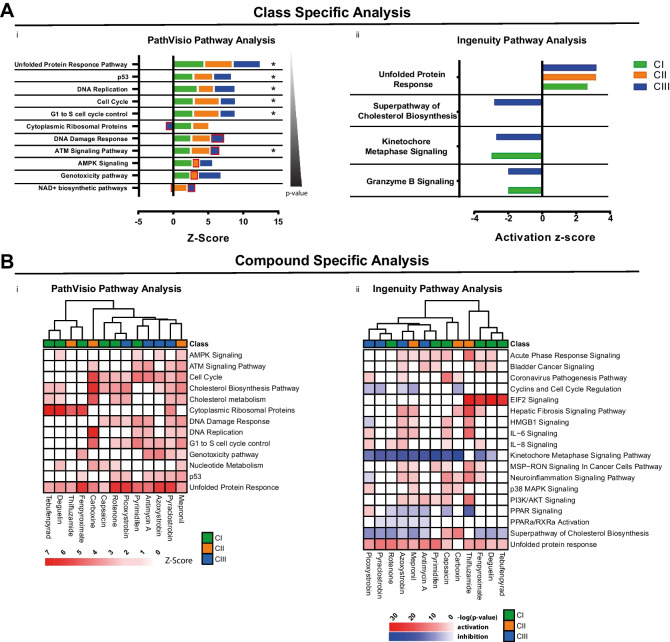
Pathway analysis. **A** Class-specific altered pathways. i. ORA analysis performed with PathVisio, starting from the class-specific inhibition response. Asterisks indicate pathways changed in the pathway analysis of the intersect gene list (Figure S2B). Cuts-off of significance include *Z*-score > 2 and permuted *p* value < 0.05. Red boxes indicate pathways below the threshold of significance. ii. ORA analysis performed with IPA, starting from the class-specific inhibition response, cuts-off of significance include absolute activation *z*-score > 2 and *p* value < 0.05. **B** Compound-specific altered pathways. Displayed pathways are induced from at least four compounds. i. Compound-specific pathway analysis performed with Pathvisio, cuts-off of significance include Z-Score > 2 and permuted *p* value < 0.05. ii. Compound-specific pathway analysis performed with IPA, cuts-off of significance include *p* value < 0.05

ORA analysis upon ETC inhibition identified the unfolded protein response (UPR) as the major altered pathway in both PathVisio with a *Z*-score of 4.43, 4.08 and 4 for CI, CII and CIII respectively, and IPA with an activation *z*-score of 2.67, 3.162 and 3.2 respectively (Fig. 4A(i–ii)). Whereas IPA ORA indicated the UPR as the only pathway changed by the three classes of inhibitors, PathVisio ORA identified in addition, alterations of the DNA damage response pathway (p53) and the cell cycle with lower *Z*-score (Fig. 4A), predominantly through the activation of *GADD45A* (Figure S2C). The same pattern of altered pathways was found for analysis of the intersect gene list (Figure S2B), suggesting that the signature of ETC inhibition is not dependent on which complex along the chain is inhibited.

Compound-specific pathway analysis confirmed the induction of the UPR by all compounds at reference concentrations using both, the PathVisio and the IPA platforms (Fig. 4B), with exception of carboxin for which the activation prediction of UPR was not significant in the IPA analysis (Fig. 4B(ii)). Beside the UPR, alteration in the cholesterol biosynthesis pathway was predicted to be affected with treatment of 9 and 11 out of 13 chemicals for PathVisio and IPA ORA respectively. Genes shared between pathways of the two platforms represent 60% and 67% of measured genes for PathVisio and IPA respectively. Interestingly, although for most compounds the prediction state indicated an inhibition of the pathway, exposure to CI inhibitor capsaicin and CII inhibitor carboxin resulted in its activation (Fig. 4B (i–ii), Figure S4A).

Moreover, both PathVisio and IPA identified changes in cell cycle related genes induced by almost all ETC inhibitor and more specifically IPA identified a downregulation of the kinetochore signalling pathway. Albeit pathway clustering did not highlight emerging compounds based on induced pathways, compound clustering, encompasses tebufenpyrad, deguelin, fenpyroximate and thifluzamide at reference concentration as inducers of the eIF2 signalling pathway (IPA) (Fig. 4B(ii)) and ribosomal protein pathway (PathVisio) (Fig. 4B(i)). Although indicated with different names in the two bioinformatic platforms, the degree of overlapping genes identifies the eIF2 signalling and ribosomal protein pathways as representation of the same response, with an overlap of 29 genes involved in ribosome synthesis: 60% and 78% of measured genes for eIF2 signalling pathway and ribosomal protein pathways respectively. Moreover, a more detailed analysis of this response, that is not limited to the single pathway genes and the applied cut-off, showed a homogeneous response within the different ETC inducers (Figure S4B).

Despite the two bioinformatic platforms rely on different databases for pathway activation predictions, both indicated a major activation of the UPR pathway. Nonetheless, the use of two different bioinformatic tools, also introduces discrepancies given by the source of knowledge provided by both platform and for which pathway analysis is largely dependent. For instance, a pathway present in one database might not be present in another database, e.g. the kinetochore signalling pathway was identified by IPA but not by PathVisio analysis, this is due to the fact that the same gene set was incorporated in the cell cycle pathway in the latter, which comprising a much larger number of genes did not produce a significant *Z*-score in the statistical analysis. In a more tedious scenario, pathways representing the same response are described with different titles in the two databases, e.g. the eIF2 signalling pathway and the ribosomal protein pathway discussed above.

Each of the two different tools provide specific information on transcripts behaviour, therefore a combined approach for the study of pathway activation that leads to robust results, is advisable. Moreover, a type of analysis that escalates different levels of complexity is necessary for a good interpretation of results.

To further evaluate the mitochondrial-specific effects at the transcriptional level, we performed a class-specific mitotoxic response analysis including only doses of compounds that impaired mitochondrial respiration (decreased OCR) without effecting viability (Figure S3A) (see “Class-specific mitotoxic response analysis” section for details). Comparison of the class-specific responses and class-specific mitotoxic responses show and overlap of significantly changed genes of 44% and 56% for CI and CIII respectively. Included in this overlap are several genes of the UPR pathway, including *DDIT3, HSPA5, PPP1R15A, ATF3, CXCL8* and *DNAJB9* as well as predominant genes such as *KRT17, GADD45A, AREG, ATF3* for both CI and CIII mitotoxic responses (Figure S3B). Pathway analysis performed with PathVisio highlighted the UPR, the cytoplasmic ribosomal protein and the genotoxicity pathways as the major changed pathways for both CI and CII classes, followed by alteration of the cholesterol biosynthesis and metabolism pathways for CIII (Figure S3C). Despite, the mitotoxic response analysis showed some degree of difference in the significant DEGs compared to the complete class-specific dataset, it confirmed the UPR (PERK/ATF4/CHOP) stress response as the main mechanism of adaptation upon inhibition of CI and CIII of the ETC.

### Upstream regulator analysis justifies pathway analysis

To identify upstream transcriptional regulators whose cascade could justify the changes experimentally observed in gene expression, an upstream regulators analysis was performed in IPA for CI, CII and CIII inhibition.

**Fig. 5:**
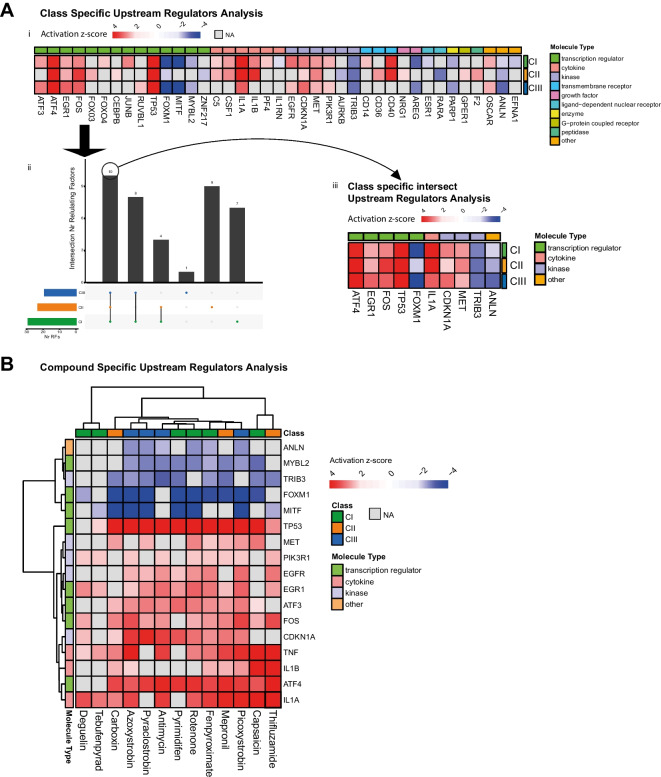
Upstream regulator analysis. **A** i. Heatmap of upstream regulators retrieved by analysis using IPA upon class-specific inhibition of CI, CII, and CIII of the ETC. Activation z-score indicate the state of prediction, positive red values for induction of the small molecule activity and blue negative values for inhibition of the activity. Cuts-off of significance include abs. activation *z*-score > 2 and BH corrected *p* value of overlap. ii. Intersection plot of the class-specific inhibition activated molecules. iii. Heatmap of the small molecules activated concomitantly from the three classes of inhibitors. **B** Heatmap of upstream regulators activated with treatment of compounds at reference concentrations and retrieved by analysis using IPA

A series of upstream regulators were identified to be activated with the treatment with one, two or all three classes of inhibitors. The predicted direction of the activation (induction or inhibition compared to basal levels), resulted to be the same for the three classes of inhibitors, again indicating a similar mechanism of action. Most of the upstream regulators predicted to be affected are transcription factors (14 out of 39), followed by cytokines and kinases (6 out of 36) (Fig. 5A(i)).

To narrow down the list of upstream regulators relevant to the ETC inhibition signature, we identified the molecules that were activated concomitantly from the three classes of treatment; the transcription factors *ATF4*, *EGR1*, *FOS* and *TP53*, are predicted to be induced, whereas the transcription factor *FOXM1* is predicted to be inhibited. The kinases *MET* is predicted to be induced as well as the kinase inhibitor *CDKN1A*, whereas *TRIB3* is predicted to be inhibited. Furthermore, general ETC inhibition induced the cytokine *IL1A* and inhibited the actin-binding protein *ANLN* (Fig. 5A(ii–iii)). The analysis of active upstream regulators upon treatment with individual compounds at reference concentrations, demonstrated a homogeneous response across inhibitors of different classes (Fig. 5B), corroborating the previous finding of a unique response for ETC inhibition which is not dependent on the complex inhibited and providing a mean of ranking the single compound based on their potency at effective concentrations.

Both class- and compounds-specific upstream regulator analysis demonstrated the activation of transcription factors involved in the previously observed pathway activation. More specifically *ATF3* and *ATF4* for UPR pathway and *TP53* for DNA damage stress responses pathway. Moreover, activation of cytokines indicates the induction of an inflammatory process beside a stress response.

### Exploring the UPR stress response pathway in RPTEC/TERT1 cells

**Fig. 6:**
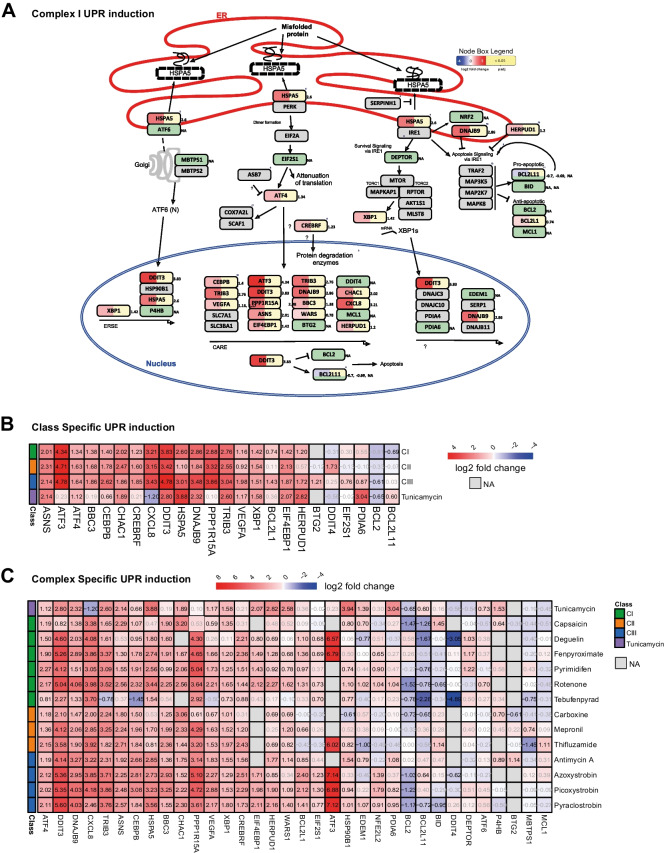
UPR response. **A** Graphical representation in PathVisio of the UPR pathway induced by CI inhibition. The log2 fold change over control is visualized on the left side of the data node boxes using a gradient from blue (-4) over white (0) to red (5). The *p* adj value is visualized on the right side of the data node boxes, yellow for *p* adj < 0.05. Full green node boxes indicate unchanged genes. Full gray node boxes indicate untested genes. **B** Heatmap comparing the log2 fold change over control of genes depicted in the UPR graphical representation that were significantly altered by at least one class of inhibitors, and the UPR inducer tunicamycin response (10 µM). **C** Heatmap comparing the log2 fold change over control of genes significantly altered in the UPR pathway induced by test compounds at reference concentrations and the UPR inducer tunicamycin (10 µM). In grey non-significant responses. NA represent excluded probes with baseMean lower than 10 reads

Pathway and upstream regulator analysis indicated the UPR as the most affected pathway by ETC inhibition. To examine the effect of ETC inhibition on the individual components of the UPR pathway, we used the graphical representation feature of PathVisio to generate a complete overview of the pathway, including the list of genes involved in the three separate branches (*ATF6*, *PERK*, *IRE1*), their links and their regulation based on the literature (Fig. 6A, Figure S5). The well-known UPR inducer tunicamycin used as positive control, induces the expression of all 3 branches of the UPR and related downstream genes including *ATF6* and the downstream chaperone *P4HB* (Fig. 6C). In contrast, although ETC inhibition upregulated many UPR genes, the transcription factor *ATF6* was not induced, nor *P4HB*, indicating a favoured response from the *PERK* and *IRE1* branches through the effect of the transcription factors *AFT4* and *XBP1* respectively (Fig. 6B, C). However, the nature of pathway analysis is that they are inherently biased to the number of entities appearing and therefore ATF6 activation cannot be ruled out, particularly since *XBP1* mRNA was induced which is under ATF6 downstream control. Comparison of significant UPR-associated genes induction indicates that the three classes of inhibitors share the same expression pattern for 2/3 of the induced genes, whereas the expression pattern was comparable to the one of tunicamycin only for about a third (12/29 for CII and 14/29 for CI and CIII) (Fig. 6B). Despite class-specific analysis demonstrated a set of UPR genes induced by the three classes of inhibitors, analysis at the compound level showed significant induction at relevant concentrations for all compounds only for *ATF4*, *DDIT3, DNAJB9* and *CXCL8*, key genes such as *HSPA5* and *XBP1* resulted activated from 12 and 11 out of 13 compounds respectively (Fig. 6C).

**Fig. 7:**
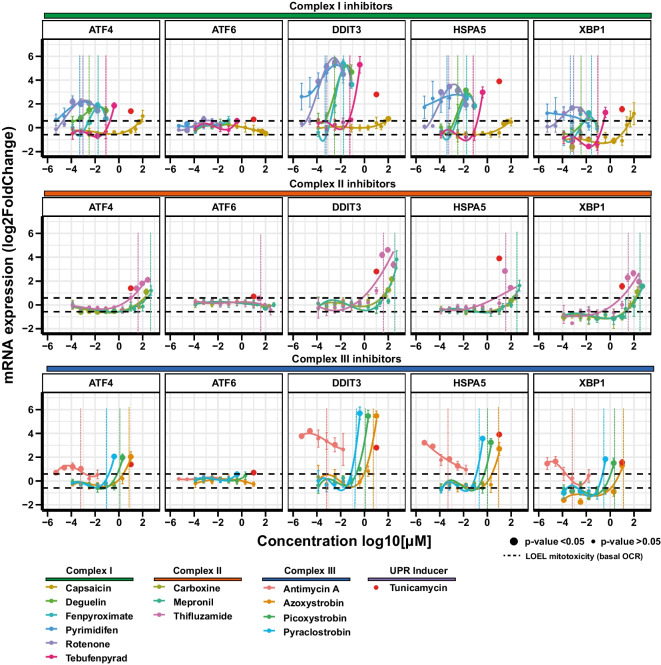
UPR dose response. Dose responses of mRNA expression of five relevant genes involved in the UPR stress response pathway. RPTEC/TERT1 cells were treated for 24 h with non-cytotoxic concentrations of inhibitors of CI, CII, and CIII of the ETC. Black dashed horizontal lines indicated the fold change cut-off (abs 1.5). Data represent the mean of 3 independent experiments ± SEM. Significant responses with *p* value < 0.05 are represented with bigger circles. Statistical significance was computed by two paired *t* test between control and treatment groups. Dashed vertical lines correspond to the onset of mitochondrial insult (basal OCR LOEL)

To evaluate the dose dependency of the major players of the UPR induction by the individual inhibitors, we assessed the concentration–response relationship for *ATF4*, *ATF6*, *DDIT3*, *HSPA5* and *XBP1* in the non-cytotoxic range. As shown previously in this study *ATF6* was not induced by reference concentrations of compounds nor by any other compound-concentration combinations, but only from tunicamycin. All compounds exhibited a dose dependent increase in UPR gene induction (Fig. 7). Most of CI inhibitors exhibited a dose dependent increase in gene induction starting at the nanomolar range and before the onset of mitochondrial toxicity (Fig. 7 vertical lines), excluding capsaicin which shows a weak induction at 100 µM. Similarly, CII inhibitors showed dose dependency starting at highest tested concentrations. Most of CIII inhibitors were active at sub-toxic concentrations at the starting point of mitochondrial insult. In contrast, antimycin A peak of activity was detected at the lowest concentration tested and the response decreased in a dose dependent manner.

The study of the dose response of UPR genes, corroborated higher potency of pyrimidifen, rotenone, deguelin, fenpyroximate and antimycin A compared to the other tested inhibitors. For those compounds induction of the biological response starts at the lowest tested concentration, two or three orders less than those necessary to induce mitochondrial toxicity. A list of BMDs and LOEL for UPRs genes is provided in Table 3.

**Table 3:**
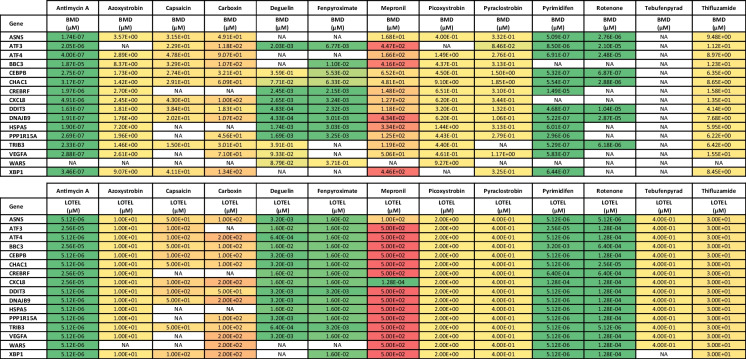
BMDs and LOELs of UPR’s genes response to test chemicals

Included genes were changed from all three classes of inhibitors. BMDs were determined in BMDExpress2 using a Exp5 parametric model to fit the dose − response curves Significance cuts − off include padj < 0.05, abs fold change > 1.5

### Relation between changes in gene transcription and protein levels

**Fig. 8:**
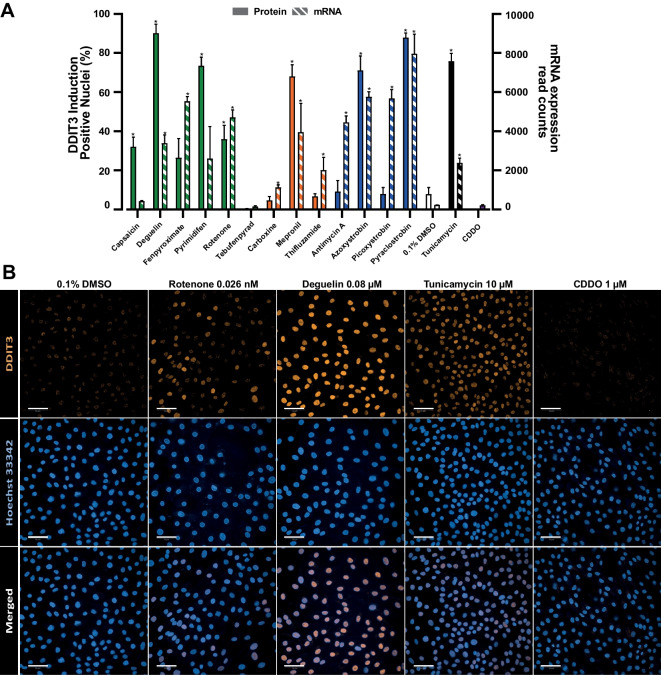
DDIT3 quantification. **A** Comparison of DDIT3 protein accumulation in the nucleus and mRNA expression in RPTEC/TERT1 treated with reference concentrations of ETC inhibitors. Protein quantification is expressed as percentage of number of positive nuclei over the total number of nuclei. Data represent the mean of 3 independent experiments ± SEM. Statistical significance was computed by two paired *t* test between control and treatment groups. Asterisks indicate a *p* value < 0.05. **B** Representative images of DDIT3 immunofluorescence in RPTEC/TERT1 of CI inhibitor deguelin and rotenone, 0.1% DMSO-treated samples, the UPR inducer tunicamycin as positive control and the Nrf2 inducer CDDO as negative control. Images were taken using confocal microscopy with × 40 water objective. Scale bars are 50 µm

To address the biological meaning of transcriptional changes upon ETC inhibitors treatment, we examined the correlation between the upregulation of the UPR’s gene *DDIT3* (CHOP) and its protein expression levels. RPTEC/TERT1 were treated for 24 h with reference concentrations of CI, CII and CIII inhibitors of the ETC, the UPR inducer tunicamycin (10 µM) as positive control and the Nrf2 inducer CDDO (1 µM) as negative control, protein expression levels were assessed through immunofluorescence (Fig. 8A, B, Figure S6). All CI inhibitors, significantly induced the accumulation of the CHOP protein in the nucleus (Fig. 8A). Although CII inhibition increased CHOP mRNA expression with treatment with all three compounds, only treatment with mepronil resulted in an increased protein level. Analogously, all CIII inhibitors increased CHOP mRNA expression, but only azoxystrobin and pyraclostrobin induced the protein expression. The discrepancy between transcripts levels and their protein domains encountered in this study, has not to be considered unusual. Different groups have studied the correlation between mRNA/protein levels, concurring that only about 40% of the mRNA abundance correlates with the protein product (Tian et al. 2004; Lundberg et al. 2010; Vogel et al. 2010; Schwanhüusser et al. 2011) and such correlation is mainly related to housekeeping genes or steady-states-conditions (Schwanhüusser et al. 2011; Liu et al. 2016). These results are not surprising given the large number of events occurring between the production of mRNAs and functional proteins, including translation, post translational modifications and protein degradation. Also, the time of onset the ATF4 response likely varies somewhat between compounds and exposure concentrations, which would additionally affect the 24 h protein expression.

### Mechanisms of oxidative stress protection upon ETC inhibition in differentiated RPTEC/TERT1 cells

**Fig. 9:**
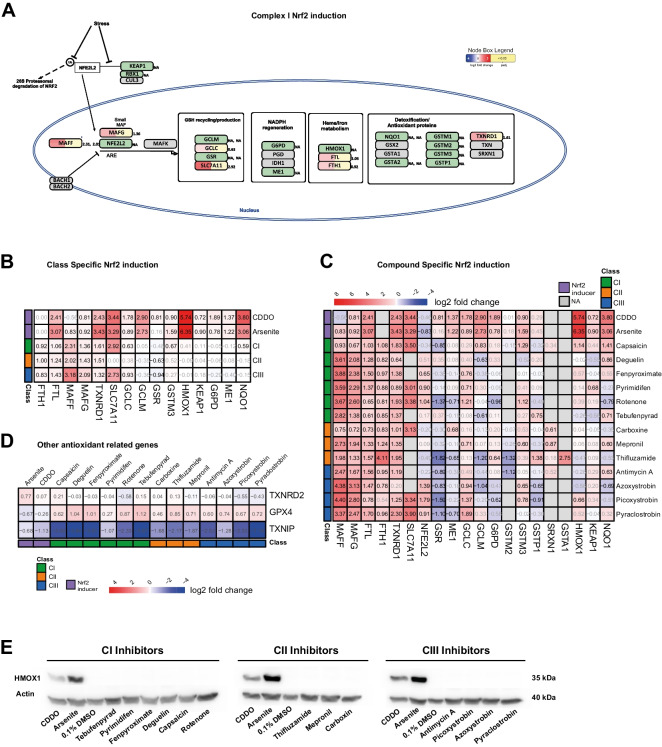
Nrf2 response. **A** Graphical representation in PathVisio of the Nrf2 pathway induced by CI inhibition. The log2 fold change over control is visualized on the left side of the data node boxes using a gradient from blue (-4) over white (0) to red (5). The *p* adj value is visualized on the right side of the data node boxes, yellow for *p* adj < 0.05. Full green node boxes indicate unchanged genes. Full gray node boxes indicate untested genes. **B** Heatmap comparing the log2 fold change over control of genes significantly altered in the Nrf2 pathway induced by CI, CII, CIII inhibitors and the Nrf2 inducers arsenite (10 µM) and CDDO (1 µM), cuts-off: abs fold change > 1.5, *p* adj < 0.05. **C** Heatmap comparing the log2 fold change over control of genes significantly altered in the Nrf2 pathway induced by test compounds at reference concentrations and the Nrf2 inducers arsenite (10 µM) and CDDO (1 µM), cuts-off: abs fold change > 1.5, *p* adj < 0.05. In grey non-significant responses. NAs represent excluded probes with baseMean lower than 10 reads. **D** Heatmap comparing the log2 fold change over control of other genes involved in the antioxidant response machinery, upon exposure of reference concentrations of compounds and the Nrf2 inducers arsenite (10 µM) and CDDO (1 µM). Cuts-off: baseMean < 10 reads. **E** Western blot of HMOX1 expression in samples treated for 24 h with representative concentrations of inhibitors of CI, CII, CIII of the ETC and inducers of the Nrf2 pathway arsenite (10 µM) and CDDO (1 µM)

**Fig. 10:**
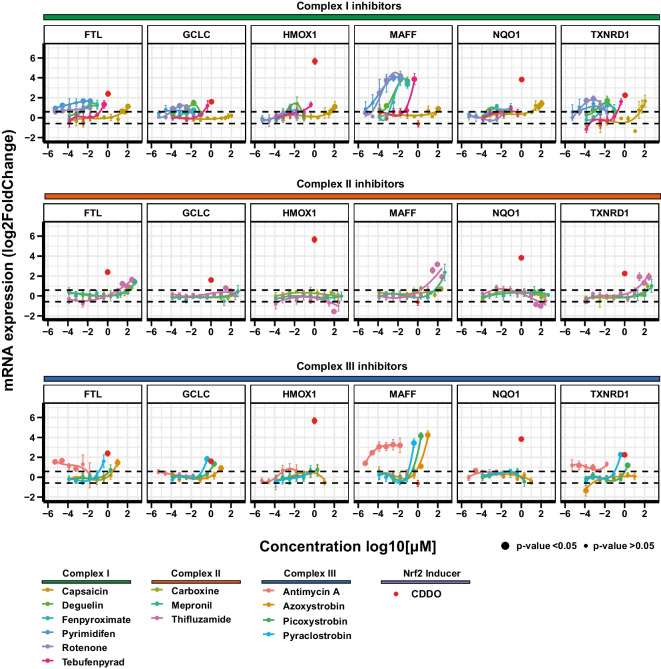
Nrf2 dose response. Dose responses of mRNA expression of relevant genes involved in the Nrf2 stress response pathway. RPTEC/TERT1 cells are treated for 24 h with non-cytotoxic concentrations of inhibitors of CI, CII, and CIII of the ETC. Black dashed lines indicated the fold change cut-off (abs 1.5). Data represent the mean of 3 independent experiments ± SEM. Significant responses with *p* value < 0.05 are represented with bigger circles. Statistical significance was computed by two paired t-test between control and treatment group

During oxidative phosphorylation, a portion of electrons (0.2/2%) leaks out the ETC to directly react with molecular oxygen to form ROS such as O_2_^−^ and H_2_O_2_. The major sites of ROS production are *I*_q_ (coenzyme-Q (CoQ) binding site) and *I*_f_ (FMN site) of CI, which produce ROS into the mitochondrial matrix, and Q_i_ site of CIII which releases electrons in both the matrix and the intermembrane space, although compared to CI the amount of ROS produced by CIII is small and can be neglected. CI inhibitors such as rotenone, inhibit the NADH-dehydrogenase complex at the level of the *I*_q_ site, blocking the regular transfer of electrons to the CoQ. Electrons are forced to return to the I_f_ site, which gets over-reduced, increasing the electron leak and the ROS production (Zhao et al. 2019). Excess of cellular ROS induces oxidative stress which triggers the Nrf2-antioxidant response element signalling pathway in the attempt to restore cellular homeostasis by enhancing the transcription of several antioxidant and detoxification proteins, the most specific being *NQO1* and *HMOX1* (Jennings et al. 2012). Interestingly, although CI inhibitors resulted to have the strongest activity in terms of transcriptional induction, pathway analysis of transcripts previously performed in this study did not highlight Nrf2 activation (Fig. 4). To investigate the effect of ETC inhibition on Nrf2’s genes, we compared the expression profiles of ETC inhibitors and Nrf2 inducers (sodium arsenite and CDDO) treated samples. The list of genes involved in the Nrf2 pathway and their expression upon CI inhibition is depicted in Fig. 9A, whereas genes of the pathway changes by either CI, CII, CIII, sodium arsenite or CDDO treatment are illustrated in Fig. 9B. Fifteen out of 31 of the genes included in the Nrf2 pathway were measured with our gene panel and 7 were induced by ETC inhibition, including: the encoding genes of ferritin’s light and heavy chain *FTL* (average log2 FC 1.24 ± 0.15) and *FTH1* (average log2 FC 0.91 ± 0.07), the transcription factors *MAFF* (average log2 FC 2.50 ± 0.50) and *MAFG* (average log2 FC 1.62 ± 0.32) and the thioredoxin reductase *TXNRD1* (average log2 FC 1.48 ± 0.12) whereas the amino acid transporter *SLC7A11* (average log2 FC 2.82 ± 0.10) and the glutathione synthesis enzyme *GCLC* (average log2 FC 0.78 ± 0.14) were only changed by CI and CIII (Fig. 9B). On the other hand, treatment with the oxidative stress inducers CDDO and sodium arsenite upregulated an additional eight genes, including the prototypical genes of the Nrf2 stress response pathway *HMOX1* (average log2 FC 6.04 ± 0.3) and *NQO1* (average log2 FC 3.42 ± 0.37). The compound-specific induction provided an overview of the compounds’ contribution to the class-specific response (Fig. 9C). Compounds clustering separated the Nrf2 inducers from the ETC inhibitors independently on their class and indicated *MAFF* as the only gene being upregulated by all ETC inhibitors. The complete concentration response curves for five representative genes on the Nrf2 pathway are depicted in Fig. 10. Neither *HMOX1* nor *NQO1* induction was found along the tested concentration range for ETC inhibitors, while *MAFF* was upregulated in a dose dependent manner, reaching 3/fourfold increase over the Nrf2 inducers. To exclude a possible transcriptional induction of *HMOX1* by ETC inhibitors occurring earlier than 24 h, we verified the presence of its protein product in samples treated with reference concentrations of compounds. We found a total lack of *HMOX1* product upon ETC inhibition, whereas the protein resulted abundant upon treatment with sodium arsenite and CDDO (Fig. 9E).

## Discussion

Chemical hazard identification and characterisation, still presents a large challenge during the early stages of the drug discovery process and for other products where humans and the environment are exposed. Animal testing remains the bedrock of chemical safety assessment, but due to species differences, complexities in addressing cellular mechanisms and ethical considerations a concerted effort is being made to transition chemical safety evaluation toward non-animal approaches. In vitro cell culture models are well utilised tools to this end representing a reductionist approach, removing higher order systems, reducing complexity, and are thus ideally suited to investigating the early stages of toxicological events. In vitro models and are valuable tools for mechanistic approaches including OCR measurements, biochemical assays, imaging and omic technologies (Jennings 2015). On a cellular level, rapid cellular adaptation to homeostatic perturbations, occurs mainly through altered gene expression via hub stress response transcription factor activation (Jennings et al. 2012). For this reason, transcriptomics is becoming a common and useful tool in toxicological studies as multiple pathways are simultaneously measured in the same sample. The current study aimed to identify transcriptional signatures associated with chemically induced ETC inhibition.

Chemical-induced mitochondrial dysfunction is gaining momentum as a previously overlooked mechanism responsible for many types of toxicological outcomes (Dykens and Will 2008). To understand the role of mitochondrial impairment in cellular toxicity, contact-inhibited human renal proximal tubular epithelial cells (RPTEC/TERT1 cells) were treated for 24 h with a range of concentrations of CI, CII and CIII inhibitors of the ETC prior to transcriptomics analysis. Pathway analysis (ORA) identified the UPR pathway as the predominant biological response to ETC inhibition. Schematic representation of the UPR pathway, including gene expression levels and key protein interaction, highlights the *PERK/*ATF4 branch of the UPR as the most highly activated.

Upon endoplasmic reticulum (ER) stress, where an accumulation of misfolded proteins is detected, the UPR pathway initiates a series of events that temporarily stop translation, activate ER-associated degradation (ERAD) and prime the cell for renewed translation via transcriptional reprogramming (Gaudette et al. 2014; Adams et al. 2019). A number of perturbations have been studied that induce ER stress including calcium perturbations, hypoxia, viral infection, specific genetic mutations, specific chemical insult, amino acid depletion and nutrient deprivation (Ron 2002; Chakrabarti et al. 2011; Adams et al. 2019; Read and Schröder 2021). The UPR is initiated by the dissociation of Bip (*HSPA5*) from three ER bound proteins, the inositol requiring enzyme 1 α/β (*IRE1*), the activating transcription factor 6 (*ATF6*) and eIF2 kinase PKR-like endoplasmic reticulum kinase (*PERK*). *IRE1* exist in two isoforms; *IRE1β* mediates translational attenuation by 28S rRNA cleavage (Iwawaki et al. 2001) and *IRE1α* activation leads to the splicing of X-box-binding protein 1 (*XBP1*) mRNA, which acts as a transcription factor (XBP1s) (Bravo et al. 2013). *ATF6* activation leads to the release of its cytosolic portion, the bZIP transcription factor ATF6N (Taouji et al. 2013). Activated PERK phosphorylates eukaryotic initiation factor 2α (*eIF2α*) which represses general translation while increasing the translation of activating transcription factor 4’s (*ATF4*) (Bravo et al. 2013; Adams et al. 2019).

*ATF4* is a basic leucine-zipper (bZIP) transcription factor, which binds to C/EBP-ATF response elements (CARE) elements in DNA leading to the expression of several downstream genes, including DNA Damage Inducible Transcript 3 (*DDIT3*, aka GADD153 and CHOP), *TRIB3*, *ATF3*, *ASNS*, *HERPUD1* and *PPP1R15A* (GADD34), all of which were induced here upon exposure to ETC inhibitors. Links between metabolic state and *ATF4* induction have been previously observed in other in vitro studies, including the observation that *ATF4* is induced upon ETC inhibition in neuronal, liver cancer cells and iPSC induced proximal tubular-like cells (Krug et al. 2014; Jennings et al. 2022; van der Stel et al. 2022). There is an established link between mitochondrial disturbances and Parkinsonian motor disease, where UPR has been implicated as a protective mechanism (Costa et al. 2020). Post-mortem studies have identified ATF4 and DDIT3 in dopaminergic neurons of Parkinson’s patients (Hoozemans et al. 2007; Esteves and Cardoso 2020). Both animal and in vitro studies of Parkinsonian motor diseases often use the complex I inhibitors rotenone and/or MPP^+^ as chemical initiators (Ryu et al. 2002; Conn et al. 2004; Tong et al. 2015; Tong et al. 2016; Gaballah et al. 2016; Costa et al. 2020). Wu and colleagues demonstrated that oral administration of rotenone (3 mg/kg/day) for 4 weeks in rats resulted in an increase in the mRNA and protein expression of *Atf4* and *Ddit3* in midbrain tissue (Wu et al. 2013), whereas Gaballah group identified an over threefold increase in *Ddit3* mRNA expression in rat brains after sub-cutaneous injection of rotenone (1.5 mg/kg/ every second day) for 21 days (Gaballah et al. 2016). Molecular studies in whole organisms also provide evidence of a link between mitochondrial perturbation and PERK/ATF4 activation. *ATF4* and downstream genes have also been found to be induced in *Drosophila Melanogaster* upon genetically induced ETC disturbance (*COX7A* knockdown) (Sorge et al. 2020). In a mouse model (p32/C1qbp-deficient mice), mitochondrial translation inhibition, induced *elF2α* phosphorylation and *ATF4* induction, inducing transcription of *ATF4* target genes (Saito et al. 2017; Sasaki et al. 2020).

Since *DDIT3* is a highly induced UPR gene, its role has been studied under various settings. A recent study, from Kaspar et al. has implicated *DDIT3* as protective in a model of mitochondrial disease (Aspartyl-tRNA synthetase (DARS) knock out) (Kaspar et al. 2021). The authors propose *DDIT3* to be protective in mitochondrial stress by preventing overactivation of *ATF4*. *DDIT3* is a basic leucine zipper transcription factor (bZIP TF) of the dimer-forming CAAT/Enhancer Binding Protein (C/EBP) family, forming homodimers with other bZIP TFs including *C/EBPβ*, *ATF3*, *MAF* and *ATF4* (Neill and Masson 2023; Osman et al. 2023). All these genes were induced in the present study by ETC inhibitors. ChiP sequencing experiments has shown that *DDIT3* overexpression, acts predominantly as a transcription repressor, but can also upregulate certain genes (Osman et al. 2023). Interestingly, *GADD45A*, highly upregulated in this study, was amongst the gene targets supressed by *DDIT3* overexpression (non-stressed conditions) (Osman et al. 2023). It is plausible that ATF4/DDIT3 heterodimers exhibits different transcriptional outcomes and indeed *GADD45A*, which promotes cell cycle inhibition, has also been previously characterised as an *ATF4* upregulated gene (Wang et al. 2015). Thus, the possible dimerisation of the *ATF4* induced C/EBP bZIP transcription factors, allows for several interactions and may be responsible for transcriptional fine tuning and temporal alterations in transcriptional outcome.

*GADD45A* is better recognised as a member of the p53 set of regulated genes. DNA damage, cell cycle and G1 to S cell cycle progression, were all predicted to be activated and all contain *GADD45A*. In addition these pathway classes include *ATM, CCND1, CDK1, -4,-6, CDKN1A, CDKN1B, E2F1, MDM2* and *TP53*. This gene set is thus likely activated through p53 activation which was also identified as an upstream regulator. Since it is highly unlikely that the compounds used cause DNA damage, this pathway is most likely activated here through energy sensing mechanisms for example AMPK (Herzig and Shaw 2018). Indeed, AMPK was identified as an overrepresented pathway. Activation of the above mentioned set of p53 causes cell cycle arrest, one of the most energy demanding cell functions. The gene *TGLN*, which encodes transgelin was also highly induced in the ETC inhibition data set. A recent study shows that transgelin a regulator of the actin cytoskeleton is induced by p53 (Tsui et al. 2019).

It was anticipated that ETC inhibition would lead to oxidative stress as this phenomenon has been previously observed in several studies (Barrientos and Moraes 1999; Nakamura et al. 2001; Li et al. 2003). However, the Nrf2 pathway was not identified as being overrepresented in overexpression analysis nor in the upstream regulator analysis. While there was an induction of some genes associated to the Nrf2 oxidative stress response pathway including *GCLC, SLC7A11, TXNRD1* and *FTL*, other prototypical Nrf2 genes such as *HMOX-1, NQO1, ME1* and *GCLM* were not affected. Interestingly, thioredoxin interacting protein (*TXNIP*), proposed to be supressed by Nrf2 (He and Ma 2012), was strongly downregulated in all conditions (Fig. 9D). TXNIP binds to and inhibits the activity of cytosolic and mitochondrial thioredoxin (TRX-1 and TRX-2 respectively) a thiol-reducing and ROS-scavenging enzyme. Thus, decreasing TXNIP would be expected to enhance thioredoxin activity and alleviate oxidative stress. Some of the genes are likely under the transcriptional regulation of other transcription factors, e.g. *SLC7A11* has also been shown to be also regulated by *ATF4* (Zgheib et al. 2018; Torrence et al. 2021). Thus, at the present time we cannot categorically conclude if the Nrf2 pathway has been impacted by the ETC inhibitors.

The downregulation of the energy-intensive process of biosynthesis of cholesterol might be closely associated with ETC inhibition. Acetyl-CoA, ATP, oxygen, and the reducing substrates NADPH and NADH must all be provided in significant amounts for the energy-intensive process of cholesterol biosynthesis to occur (Luo et al. 2019). The low energy conditions derived from the ETC inhibition may limit the availability of all the substrates necessary for the synthesis of cholesterol (Shi and Tu 2015).

In conclusion, the ETC inhibitors had a profound effect on transcriptional regulation of contact-inhibited RPTEC/TERT1 cells, often at concentrations prior to those where inhibition of oxygen consumption was detected. The cells are thus highly sensitive to even modest decreases in mitochondrial respiration and/or ATP production. While complex I and complex III inhibitors were more potent than complex II inhibitors the pathways induced were similar. The ATF4 arm of the unfolded protein response was the most predominant transcriptional pathway activated, followed by p53 and cholesterol biosynthesis. Taken together with other studies it is likely that the ATF4 pathway is a primary sensor and adaptor of mitochondrial dysfunction. More work will be required to delineate this pathway further and determine the roles of the individual players.

### Supplementary Information

Below is the link to the electronic supplementary material.

Figure S1. Probes comparison. Fold change over control of different probes mapping the same gene from panels v2.0 and v2.2 used in experiment 1 and experiment 2 respectively upon treatment with 0.000128 μM antimycin A. 115 probes with similarity threshold of log2 fold change SDp < 0.1 were added to the class specific gene list additionally to common probes between the two panels. Row names: gene symbol v2.0_robe of v2.0;gene symbol v2.2_probe of v2.2. (PNG 242 kb)

High resolution image (EPS 1080 kb)

Figure S2. **A)** OCR and Viability tunicamycin. Dose dependent effect on basal and maximal respiration rates extrapolated from the mitostress assay using the Seahorse analyzer (pmol/min/well) and on viability my means of resazurin reduction (RFU/well) upon 24 h exposure to a range of concentrations of the UPR inducer tunicamycin. Vertical dotted line represents the concentration used in the transcriptomic analysis (10 μM). Data represent the mean of 2 independent experiments with three technical replicates each ± SEM expressed as percentage of vehicle treated control samples (0.1% DMSO). Statistical significance was computed by one-way ANOVA followed by Dunnett’s multiple comparisons posttest. Asterisks indicate a p-value <0.05. **B)**. Pathway analysis of intersect gene list. ORA analysis performed with PathVisio of the genes changed concomitantly from the three classes of inhibitors (318), cuts-off of significance include absolute Z-Score > 2 and permuted p-value < 0.05. **C)** Class Specific p53 induction. Heatmap comparing the log2 fold change over control of genes part of the p53 pathway induced by Class I, II and III, the Nrf2 inducers arsenite (10 μM) and CDDO (1 μM), and the UPR inducer tunicamycin (10 μM). In grey values of abs fold change below 1.5. (PNG 237 kb)

High resolution image (EPS 1.16 MB)

Figure S3. Mitotoxic response. A) Reduced version of Fig. 1 of the main text including only compounds that reduce OCR in viable conditions. Mitotoxic concentrations in red boxes have been used to perform the Mitotoxic set analysis. B) Volcano plots of CI and CIII Mitotoxic response. C) ORA analysis performed with PathVisio, starting from CI and CIII Mitotoxic response. Cuts-off of significance include Z-Score > 2 and permuted p-value < 0.05. (PNG 321 kb)

High resolution image (EPS 3.23 MB)

Figure S4. PathVisio and IPA ORA comparison. **A)** Heatmap comparing the log2 fold change over control of genes shared by the two cholesterol biosynthesis pathways (IPA-PathVisio) upon exposure to reference concentrations of test compounds. **B)** Heatmap comparing the log2 fold change over control of genes shared by the IPA eIF2 signalling and PathVisio ribosomal proteins pathways upon exposure to reference concentrations of test compounds. (PNG 256 kb)

High resolution image (EPS 1297 kb)

Figure S5. CI and CIII UPR response. Graphical representation in PathVisio of the UPR pathway induced by CII **(A)** and CIII **(B)** inhibition. The log2 fold change over control is visualized on the left side of the data node boxes using a gradient from blue (-4) over white (0) to red (5). The p-adj value is visualized on the right side of the data node boxes, yellow for p-adj < 0.05. Full green node boxes indicate unchanged genes. Full gray node boxes indicate untested genes. (PNG 927 kb)

High resolution image (EPS 3589 kb)

Figure S6. DDIT3 protein expression. Representative images of DDIT3 immunofluorescence in RPTEC/TERT1 treated with the inhibitors not represented in Figure 8 B. Images were taken using confocal microscopy with 40X water objective. Scale bars are 50 μm. (PNG 4990 kb)

High resolution image (EPS 106034 kb)

Table S1. Compounds and relative concentrations used in the two TempO-Seq experiments. (XLSX 11 kb)

Table S2 Complete list of probes of panel v2.0 and panel V2.2 including probe IDs, gene IDs and genes’ information. (XLSX 526 kb)

Table S3. Class specific gene list of the 2907 genes used in the class comparison transcriptomic analysis in this study, genes symbols are relative to panel v2.2. (XLSX 33 kb)

Table S4. List of pathways included in the subset database used for pathway analysis with PathVisio and their source. Pathways with source “in house drawn” were assembled according to literature research and their list of genes. Pathways with source “wikipathways” refer to wikipathways-20200610-gpml-Homo sapiens. (XLSX 17 kb)

Table S5. Table of raw counts per gene per sample of experiments 1 and 2 as provided from BioClavis, with relative annotation files. (XLSX 7026 kb)

Table S6. Table of gene lists referred to Fig. 4 B, including FoldChange, baseMean and p-adj information of CI, CII and CIII significant unique expression (49, 121 and 266 genes respectively), genes significantly changed by 2 classes of genes (27+53+197, CI_CII_CIII_paired_genes) and the intersect gene list (318 genes). Significance cuts-off are: baseMean> 10, abs FoldChange > 1.5, p-adj < 0.05. (XLSX 140 kb)

## Data Availability

Datasets on which the conclusions of the paper rely, are presented in the additional supporting files Table S5.

## Abbreviations

ACC: Activity concentrations at cutoff
BMD: Benchmark dose
CI: Complex I
CII: Complex II
CIII: Complex III
DEG: Differentially expressed genes
EAD: Equivalent administered dose
ETC: Electron transport chain
IVIVE: In vitro-in vivo extrapolation
LD50: Lethal dose of 50%
LOEL: Lowest observed effective level
MPP^+^: 1-Methyl-4-phenyl-pyridinium
NAM: New approach methodology
NOEL: No observable effect level
OCR: Oxygen consumption rates
OPERA: Open (Quantitative) Structure–activity/property Relationship App
ORA: Over representation analysis
ROS: Reactive oxygen species
UPR: Unfolded protein response
PoD: Point of departure
