# Supplementary figures and images for: Transcriptional landscape of mitochondrial electron transport chain inhibition in renal cells
Source: Cell Biol Toxicol. 2023 Jun 23;39(6):3031–59. doi: 10.1007/s10565-023-09816-7 (PMC10693540; doi:10.1007/s10565-023-09816-7)

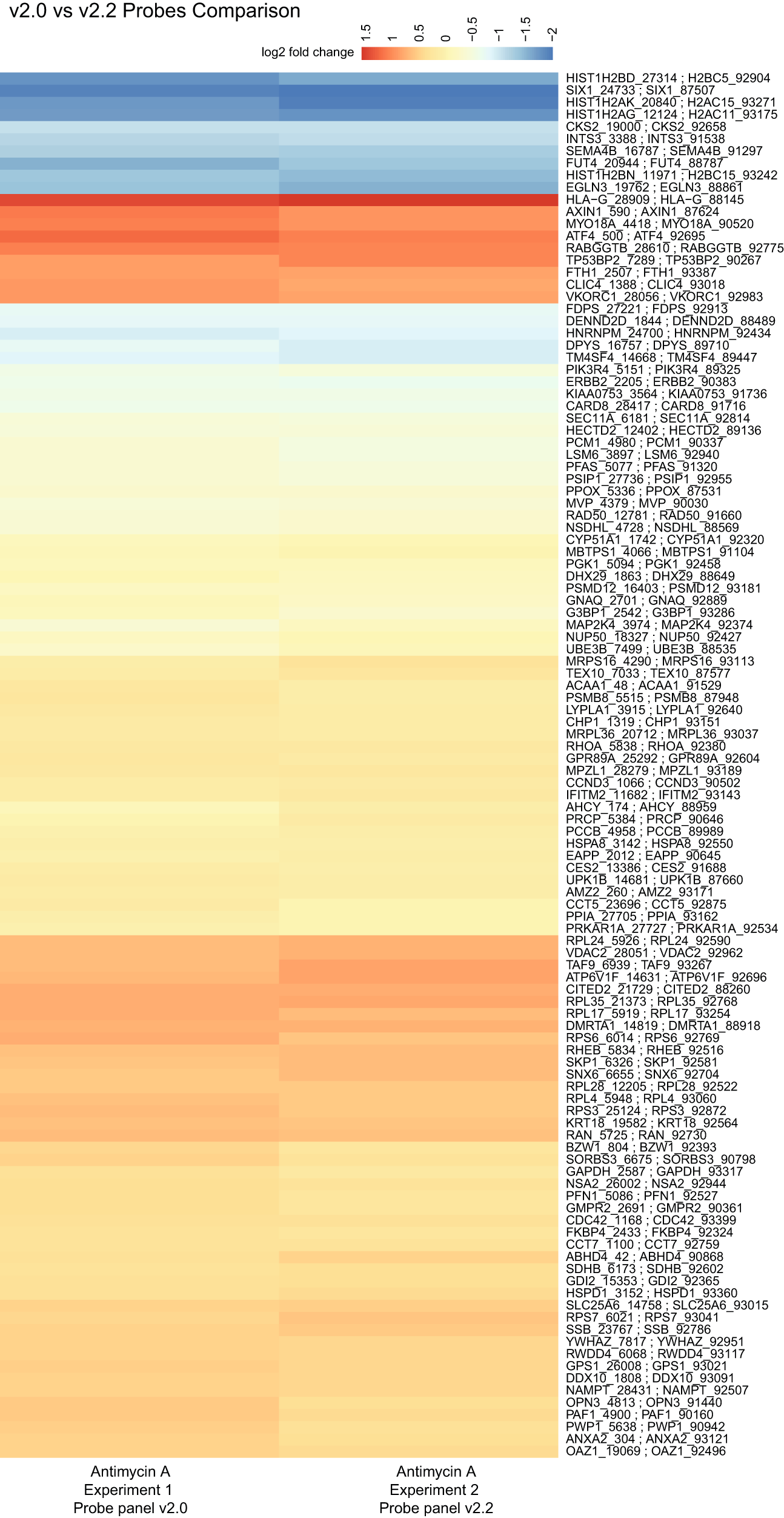

Supplement: Supplementary file 1 — Figure S1. Probes comparison. Fold change over control of different probes mapping the same gene from panels v2.0 and v2.2 used in experiment 1 and experiment 2 respectively upon treatment with 0.000128 μM antimycin A. 115 probes with similarity threshold of log2 fold change SDp < 0.1 were added to the class specific gene list additionally to common probes between the two panels. Row names: gene symbol v2.0_robe of v2.0;gene symbol v2.2_probe of v2.2. (PNG 242 kb) [file 10565_2023_9816_Fig11_ESM.png]

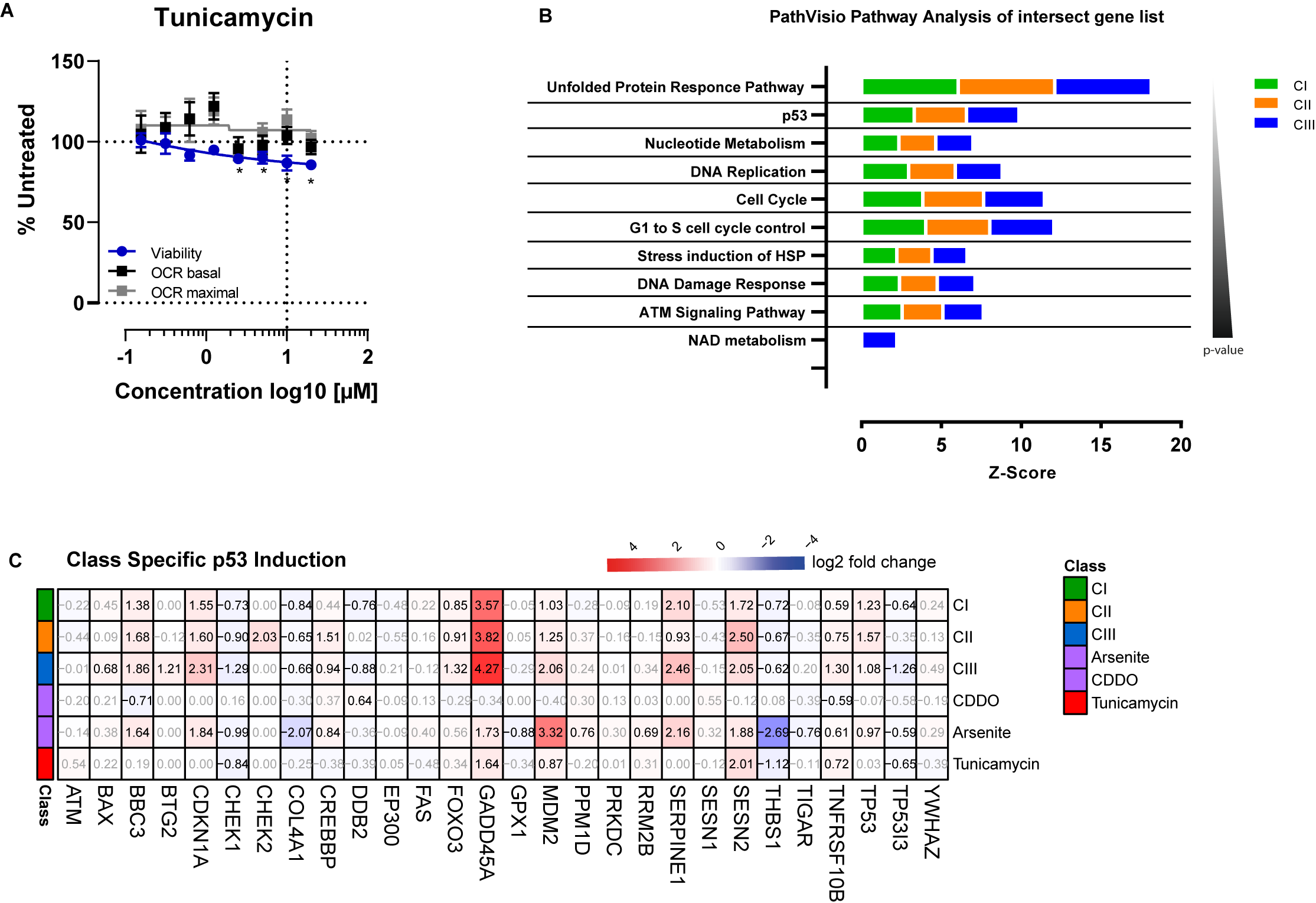

Supplement: Supplementary file 3 — Figure S2. A) OCR and Viability tunicamycin. Dose dependent effect on basal and maximal respiration rates extrapolated from the mitostress assay using the Seahorse analyzer (pmol/min/well) and on viability my means of resazurin reduction (RFU/well) upon 24 h exposure to a range of concentrations of the UPR inducer tunicamycin. Vertical dotted line represents the concentration used in the transcriptomic analysis (10 μM). Data represent the mean of 2 independent experiments with three technical replicates each ± SEM expressed as percentage of vehicle treated control samples (0.1% DMSO). Statistical significance was computed by one-way ANOVA followed by Dunnett’s multiple comparisons posttest. Asterisks indicate a p-value <0.05. B). Pathway analysis of intersect gene list. ORA analysis performed with PathVisio of the genes changed concomitantly from the three classes of inhibitors (318), cuts-off of significance include absolute Z-Score > 2 and permuted p-value < 0.05. C) Class Specific p53 induction. Heatmap comparing the log2 fold change over control of genes part of the p53 pathway induced by Class I, II and III, the Nrf2 inducers arsenite (10 μM) and CDDO (1 μM), and the UPR inducer tunicamycin (10 μM). In grey values of abs fold change below 1.5. (PNG 237 kb) [file 10565_2023_9816_Fig12_ESM.png]

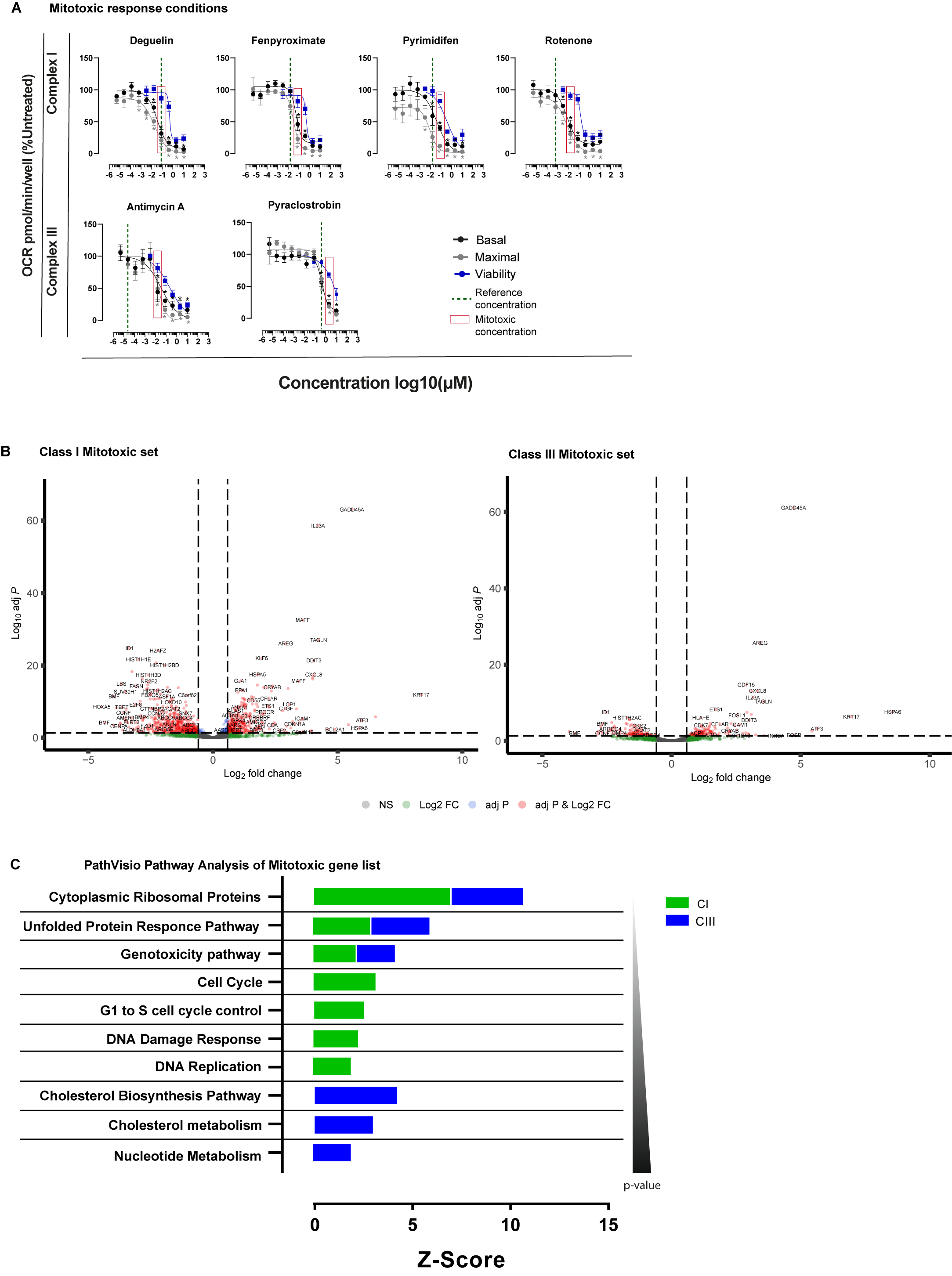

Supplement: Supplementary file 5 — Figure S3. Mitotoxic response. A) Reduced version of Fig. 1 of the main text including only compounds that reduce OCR in viable conditions. Mitotoxic concentrations in red boxes have been used to perform the Mitotoxic set analysis. B) Volcano plots of CI and CIII Mitotoxic response. C) ORA analysis performed with PathVisio, starting from CI and CIII Mitotoxic response. Cuts-off of significance include Z-Score > 2 and permuted p-value < 0.05. (PNG 321 kb) [file 10565_2023_9816_Fig13_ESM.png]

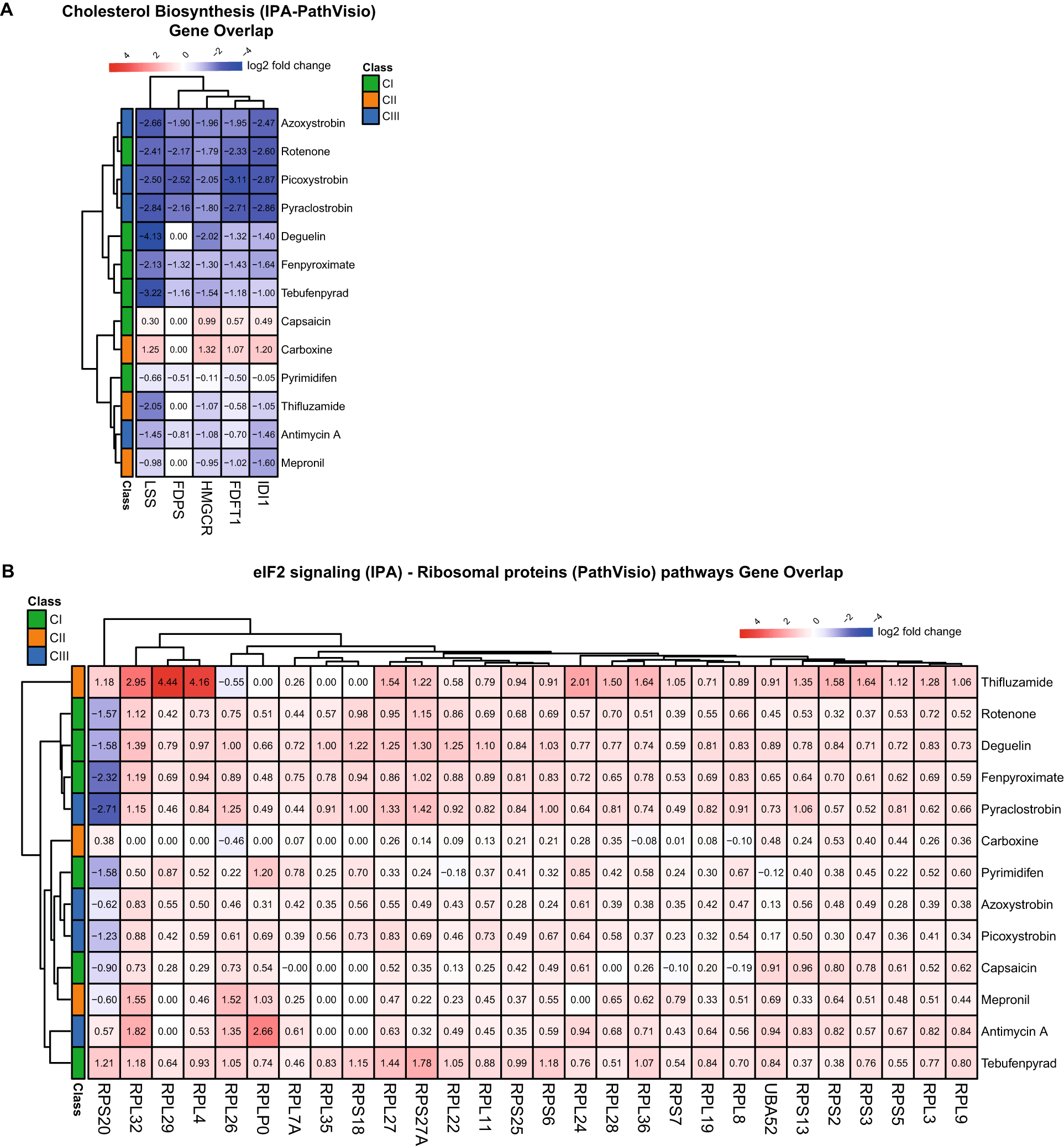

Supplement: Supplementary file 7 — Figure S4. PathVisio and IPA ORA comparison. A) Heatmap comparing the log2 fold change over control of genes shared by the two cholesterol biosynthesis pathways (IPA-PathVisio) upon exposure to reference concentrations of test compounds. B) Heatmap comparing the log2 fold change over control of genes shared by the IPA eIF2 signalling and PathVisio ribosomal proteins pathways upon exposure to reference concentrations of test compounds. (PNG 256 kb) [file 10565_2023_9816_Fig14_ESM.png]

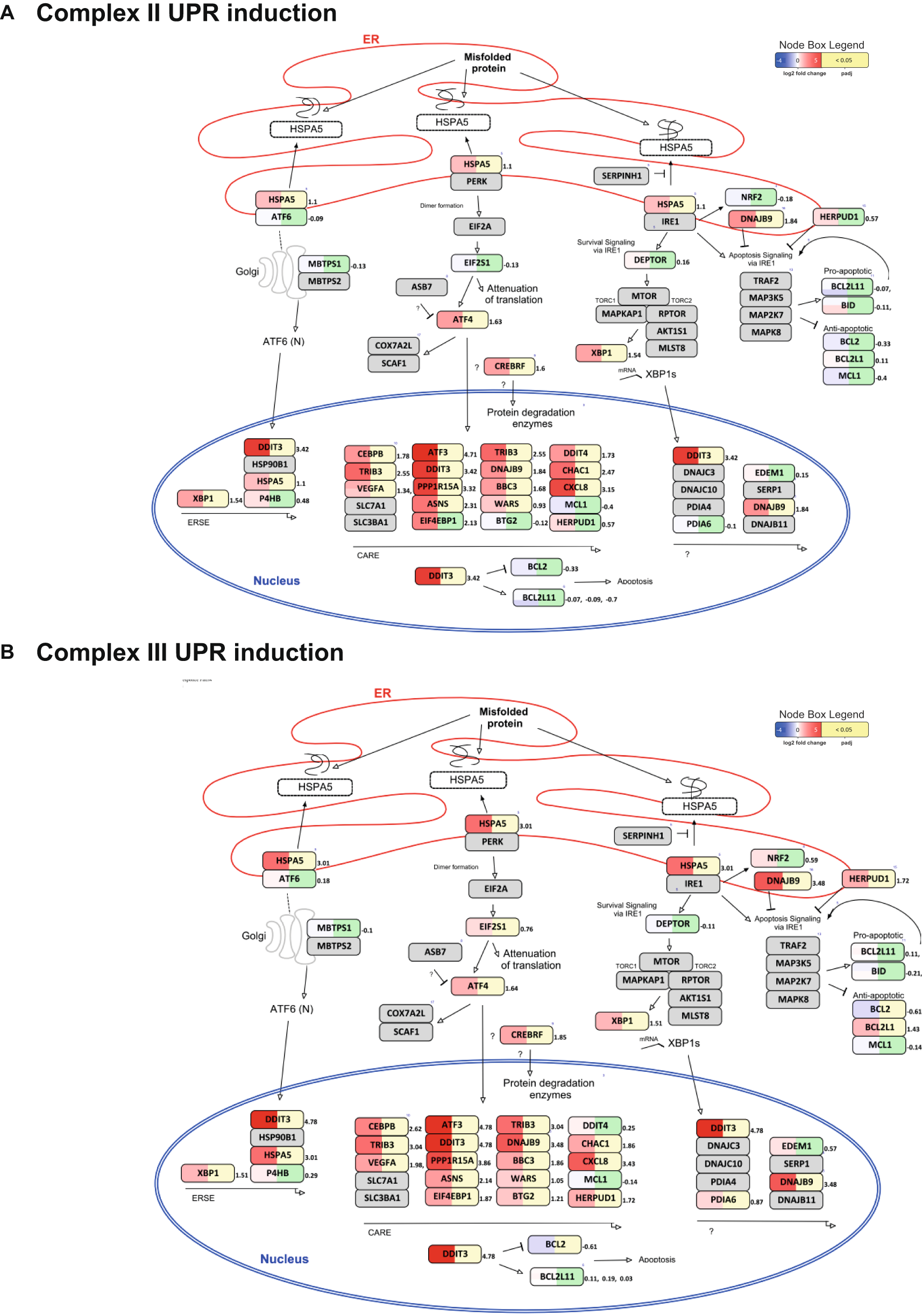

Supplement: Supplementary file 9 — Figure S5. CI and CIII UPR response. Graphical representation in PathVisio of the UPR pathway induced by CII (A) and CIII (B) inhibition. The log2 fold change over control is visualized on the left side of the data node boxes using a gradient from blue (-4) over white (0) to red (5). The p-adj value is visualized on the right side of the data node boxes, yellow for p-adj < 0.05. Full green node boxes indicate unchanged genes. Full gray node boxes indicate untested genes. (PNG 927 kb) [file 10565_2023_9816_Fig15_ESM.png]

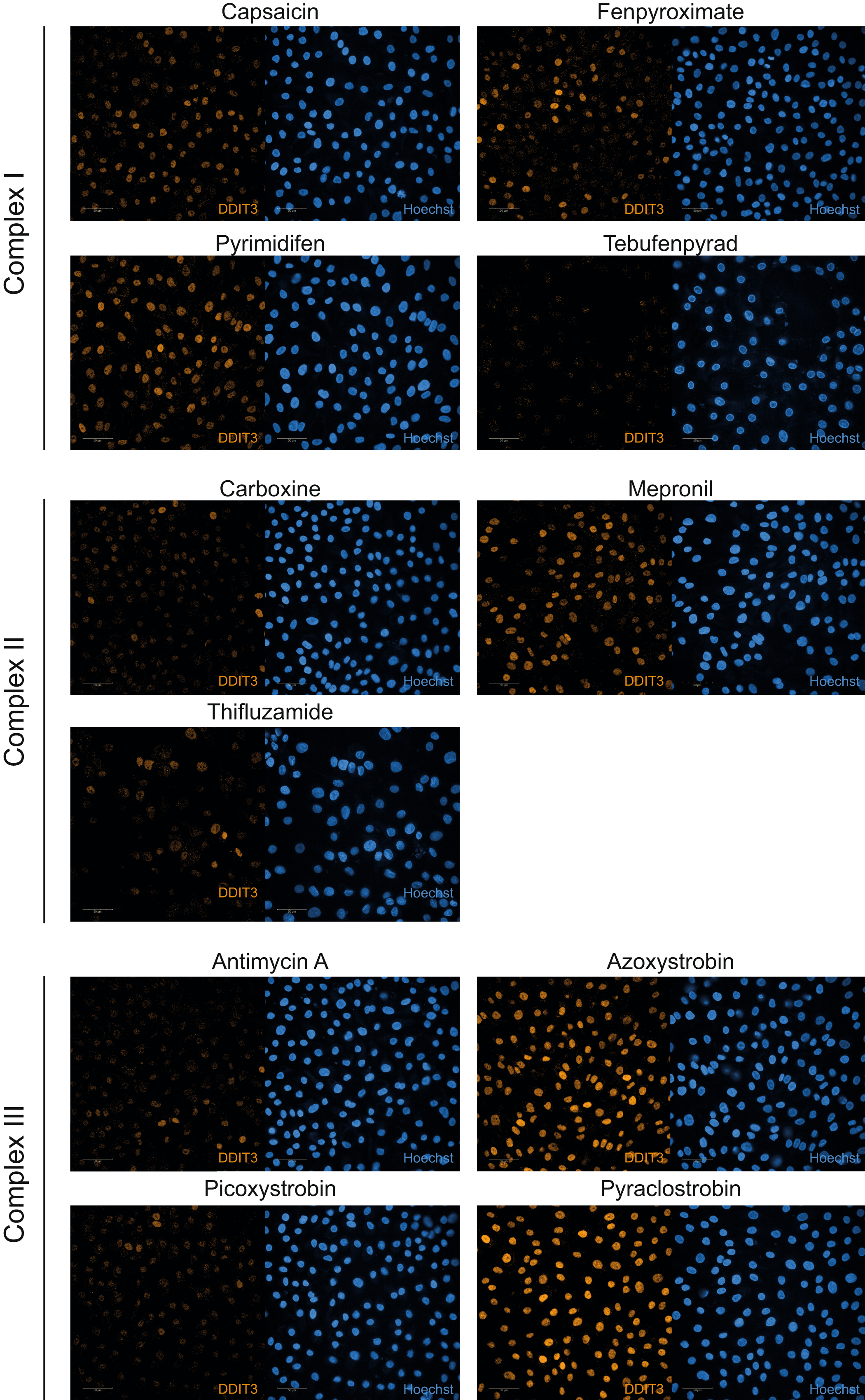

Supplement: Supplementary file 11 — Figure S6. DDIT3 protein expression. Representative images of DDIT3 immunofluorescence in RPTEC/TERT1 treated with the inhibitors not represented in Figure 8 B. Images were taken using confocal microscopy with 40X water objective. Scale bars are 50 μm. (PNG 4990 kb) [file 10565_2023_9816_Fig16_ESM.png]
